# Novel potent azetidine-based compounds irreversibly inhibit Stat3 activation and induce antitumor response against human breast tumor growth *in vivo*

**DOI:** 10.1016/j.canlet.2022.215613

**Published:** 2022-03-09

**Authors:** Peibin Yue, Yinsong Zhu, Christine Brotherton-Pleiss, Wenzhen Fu, Nagendra Verma, Jasmine Chen, Kayo Nakamura, Weiliang Chen, Yue Chen, Felix Alonso-Valenteen, Simoun Mikhael, Lali Medina-Kauwe, Kathleen M. Kershaw, Maria Celeridad, Songqin Pan, Allison S. Limpert, Douglas J. Sheffler, Nicholas D. P. Cosford, Stephen L. Shiao, Marcus A. Tius, Francisco Lopez-Tapia, James Turkson

**Affiliations:** a Department of Medicine, Division of Medical Oncology, Cedars-Sinai Medical Center, 8700 Beverly Blvd, Los Angenes, CA, 90048, USA; b Cancer Biology Program, Cedars-Sinai Cancer, Cedars-Sinai Medical Center, 8700 Beverly Blvd, Los Angeles, CA, 90048, USA; c Cancer Biology Program, University of Hawaii Cancer Center, 701 Ilalo St, Honolulu, HI, 96813, USA; d Department of Chemistry, University of Hawaii, Manoa, 2545 McCarthy Mall, Honolulu, HI, 96825, USA; e Department of Biomedical Sciences, Cedars-Sinai Medical Center, 8700 Beverly Blvd, Los Angeles, CA, 90048, USA; f Department of Radiation Oncology, Cedars-Sinai Medical Center, 8700 Beverly Blvd, Los Angeles, CA, 90048, USA; g Cell and Molecular Biology of Cancer Program, Cancer Center, Sanford Burnham Prebys Medical Discovery Institute, 10901 N. Torrey Pines Rd, La Jolla, CA, 92037, USA; h W. M. Keck Proteomics Laboratory, University of California, Riverside, CA, 92521, USA

**Keywords:** Signal transducer and activator of transcription, Small-molecule inhibitors, Covalent modification, Antitumor cell effects, Tumor growth inhibition

## Abstract

Signal transducer and activator of transcription (Stat)3 is a valid anticancer therapeutic target. We have discovered a highly potent chemotype that amplifies the Stat3-inhibitory activity of lead compounds to levels previously unseen. The azetidine-based compounds, including H172 (**9f)** and H182, irreversibly bind to Stat3 and selectively inhibit Stat3 activity (IC_50_ 0.38–0.98 μM) over Stat1 or Stat5 (IC_50_ > 15.8 μM) *in vitro*. Mass spectrometry detected the Stat3 cysteine peptides covalently bound to the azetidine compounds, and the key residues, Cys426 and Cys468, essential for the high potency inhibition, were confirmed by site-directed mutagenesis. In triple-negative breast cancer (TNBC) models, treatment with the azetidine compounds inhibited constitutive and ligand-induced Stat3 signaling, and induced loss of viable cells and tumor cell death, compared to no effect on the induction of Janus kinase (JAK)2, Src, epidermal growth factor receptor (EGFR), and other proteins, or weak effects on cells that do not harbor aberrantly-active Stat3. H120 (**8e**) and H182 as a single agent inhibited growth of TNBC xenografts, and H278 (hydrochloric acid salt of H182) in combination with radiation completely blocked mouse TNBC growth and improved survival in syngeneic models. We identify potent azetidine-based, selective, irreversible Stat3 inhibitors that inhibit TNBC growth *in vivo*.

## 1. Introduction

Signal transducer and activator of transcription (Stat) protein family are cytoplasmic transcription factors that respond to cellular stimulation by cytokines and growth factors to promote cell growth and differentiation, inflammation, and immune responses [1]. Classically, upon cytokine and growth factor binding to their cognate receptors, the Stat proteins are activated via the phosphorylation of a critical tyrosine (Tyr, Y) residue by growth factor receptor Tyr kinases, Janus Kinases (JAKs) or Src family kinases. Phosphorylation in turn drives the dimerization of two Stat monomers through a reciprocal phospho-Tyr-Src Homology 2 (SH2) domain interaction. Stat:Stat dimers then translocate to the nucleus and bind to specific DNA-response elements in target gene promoters to regulate gene transcription. By functionally controlling gene expression, the Stat proteins regulate fundamental cellular processes [1].

Unlike classical Stat signaling, which is transient in non-transformed cells, the aberrant-activation of Stat3 occurs in malignant transformation and is implicated in breast and many other human cancers [2]. Aberrantly-active Stat3 promotes tumor progression in part through its direct dysregulation of gene expression and cross-talks with other key proteins, including nuclear factor (NF)-κB, leading to uncontrolled growth and survival of tumor cells, enhanced tumor angiogenesis, and tumor metastasis [2,3]. Stat3 functioning in immune cells in the tumor microenvironment further suppresses tumor immune surveillance [2,3] to promote tumor progression. On-going research continues to uncover unconventional pathways that are regulated by Stat3 to control cell survival and cell death, including the reports that Stat3 is localized in the mitochondria [4] and in the endoplasmic reticulum (ER) to regulate calcium flux [5].

Although Stat3 is a validated target for the discovery of novel anti-cancer drugs [3,6], the discovery and development of potent small molecule inhibitors has proved to be a significant challenge. As a result, to date, no small molecule direct Stat3 inhibitors are in clinical use. Nearly all the current inhibitors of Stat3 are low in potency (micro-molar) [3,7], except the recently reported PROTAC-Stat3 inhibitors, including SD-36, which degrades cellular Stat3, with nanomolar potency and inhibits tumor growth [8,9]. Moreover, most of the current Stat3 inhibitors have unclear mechanisms of inhibition of Stat3 activity, likely indirectly blocking Stat3 phosphorylation via Tyr kinases [3,7]. Most notably, there has been minimal focus on the studies that shed light on how Stat3 inhibitors alter the dynamics of Stat3 processing in tumor cells.

Systematic, hypothesis-driven medicinal chemistry efforts using the previously reported carboxylic acid-based small molecule Stat3 inhibitor leads [10–14] have led to the discovery of a new generation of highly potent and selective Stat3 inhibitors, including H120 (**8e**), H105 (**8f**), H172 (**9f)** [15], and H182. The azetidine compound series irreversibly bind to Stat3 and preferentially and directly inhibit Stat3 DNA-binding activity *in vitro*, as exemplified by H172 and H182, with IC_50_ values of 0.38–0.98 μM, and H120 and H105, with IC_50_ values of 1.75–2.07 μM. Treatment of triple negative breast cancer (TNBC) cells with the compounds inhibited both constitutive and ligand-induced Stat3 activation, and perinuclear aggregation, which led to tumor cell death. Oral or intra-peritoneal delivery of H120 or H182 as a single agent inhibited human TNBC xenografts growth, and H278 (H182 hydrochloric acid salt) combined with radiation therapy completely blocked tumor growth and prolonged survival in mouse TNBC syngeneic models that harbor aberrantly-active Stat3.

## 2. Materials and Methods

### 2.1. Chemical synthesis of H182 and H172

Synthesis and detailed characterization of H182 and H172 are described in Supplementary Information, “Methods”.

### 2.2. Cells and reagents

Mouse fibroblasts transformed by v-Src (NIH3T3/v-Src) or over-expressing the human epidermal growth factor (EGF) receptor (NIH3T3/hEGFR), human breast cancer cell line (MDA-MB-231), the immortalized normal human breast epithelial cell line (MCF-10A) have been previously reported [12,13]. Human breast cancer cells, MCF-7, MDA-MB-468, and HCC1937 were purchased from American Type Culture Collection (ATCC) on February 12, 2020, August 26, 2020, and January 7, 2021, respectively, with authentication and mycoplasma-free certifications. The normal mesothelial cells were obtained from Dr. Michele Carbone and previously reported [16], and the murine breast tumor line (E0771) was purchased from CH3 Biosystems (Amherst, NY) and has been previously reported [17] The E0771 cells were authenticated by short tandem repeat profiling, and species-specific PCR evaluation was performed to rule out any contamination by rat, human, hamster or monkey cells. Mycoplasma tests were conducted on MDA-MB-231 and MCF-10A by IDEXX BioAnalytics (Columbia, MO) in Dec 2019 and both were negative, E0771 cells were screened and certified to be free of pathogens (Mycoplasma, Theiler’s Murine Encephalomyelitis Virus Pneumonia Virus of Mice, Minute Virus of Mice, Mouse Hepatitis Virus, Mouse Parvovirus, Corynebacterium bovis and Sendai Virus), while tests have not been performed on the human mesothelial cells. MCF-10A cells were grown in Dulbecco’s modified Eagle’s medium (DMEM)/F12 with 5% horse serum plus EGF (20 ng/ml), insulin (10 μg/ml), hydrocortisone (0.5 mg/ml), and 100 ng/ml cholera toxin, the mesothelial cells were cultured in DMEM containing 20% fetal bovine serum (FBS) and 1% Penicillin/Streptomycin [16], and the E0771 cells were grown in DMEM with 10% FBS [17]. All other cells were cultured in DMEM plus 10% heat-inactivated FBS. Antibodies against Stat3, pY705-Stat3, pS727-Stat3, pY1173EGFR, EGFR, pY1007/1008JAK2, JAK2, pY416Src, Src, Shc, p-Shc, pS473Akt, Akt, pT202/Y204Erk1/2 (p44/42), Erk1/2, PARP, caspase 3, c-Myc, survivin, β-actin, and α/β-tubulin were purchased from Cell Signaling Technology, Inc. (Danvers, MA), with the exception of VEGF, and GAPDH antibodies, which were purchased from Santa Cruz Biotechnology (Dallas, Texas). Cisplatin and docetaxel were purchased from Sigma-Aldrich (St. Louis, MO).

### 2.3. Nuclear extract preparation, gel shift assays, and densitometric analysis

These studies were carried out as previously described [11–13] using the ^32^P-labeled oligonucleotide probes, hSIE (high affinity sis-inducible element from the *c*-*fos* gene, m67 variant, 5^′^-AGCTTCATTTCCCGTAAATCCCTA) that binds Stat1 and Stat3 [11,12] and the mammary gland factor element (MGFe) from the bovine β–casein gene promoter (sense strand, 5^′^-AGATTTCTAGFAATTCAA) that binds Stat5 and Stat1 [11,12]. Details are presented in Supplementary Information, “Methods”.

### 2.4. Luciferase reporter assay

These studies were performed as previously reported [10–12,18] using the v-Src-transformed NIH3T3/v-Src fibroblasts [10–13,18]. Details are presented in Supplementary Information, “Methods”.

### 2.5. Site directed mutagenesis to create Stat3 mutants

The prokaryotic pET28-hStat3(127–711)−6 × His plasmid construct that contains the DNA fragment coding for human Stat3 protein residues 127–711 (referring to NM_139276.3) with a C-terminal 6 × His tag, which is flanked by the restriction sites NdeI and XhoI, was kindly provided by Dr. Yuan Chen at the University of California, San Diego [19]. This construct was used to generate the mutants, Stat3C328A, Stat3C426A, Stat3C468A, and Stat3C542S, as described in detail in Supplementary Information “Methods”.

### 2.6. Protein expression and purification

The expression of un-phosphorylated and tyrosine phosphorylated His-tagged recombinant wtStat3 and mutant Stat3 proteins in BL21 (DE3) bacterial cells is described in detail in Supplementary Information “Methods”.

### 2.7. SDS-PAGE/Western blotting analysis

These studies were performed as previously described [11–13]. Details are presented in Supplementary Information, “Methods”.

### 2.8. Cell viability assays

CyQuant assay was performed to evaluate compounds as previously reported [11–13]. Details are presented in Supplementary Information, “Methods”.

### 2.9. Soft-agar colony formation and clonogenic survival assays

These studies were performed as previously reported [11–13]. Details are presented in Supplementary Information, “Methods”.

### 2.10. Confocal microscopy studies

These studies were performed as previously reported [18]. Details are presented in Supplementary Information, “Methods”.

### 2.11. Fluorescence polarization assay (FP)

These studies were conducted as previously reported [11,12] using the labeled phospho-peptide, 5-carboxyfluorescein GpYLPQTV-NH_2_ (where pY represents phospho-Tyr) as probe and purified recombinant Stat3, with some modifications. Details are presented in Supplementary Information, “Methods”.

### 2.12. Isothermal titration calorimetry (ITC)

The ITC experiment was carried out as previously described [15,20]. Details are provided in Supplementary Information, “Methods”.

### 2.13. Wound healing assay for migration

Studies were performed as previously reported [11–13]. Details are presented in Supplementary Information, “Methods”.

### 2.14. Annexin V binding/apoptosis and flow cytometric analysis

For apoptosis analysis, annexin V/propidium iodide (PI) staining was performed. Details are presented in Supplementary Information, “Methods”.

### 2.15. Assessment of physicochemical properties

Solubility, human (HLM) and mouse liver microsomal (MLM), and plasma stability studies were performed by Eurofins Cerep Panlabs and Eurofin Discovery Services (www.eurofins.com/PharmaDiscovery), while protein binding studies were performed by Sanford Burnham Prebys Chemical Biology & Drug Discovery Core using standard protocols.

### 2.16. Mass-spectrometry study for the detection of covalently-bound peptide adducts

Mass Spectrometry studies to detect the peptides bound to Stat3 inhibitors are described in detail in Supplementary Information “Methods”.

### 2.17. Mice and in vivo tumor studies

Mice were housed in specific pathogen-free conditions in the animal facility. All animal experiments were conducted in accordance with the recommendations in the Guide for the Care and Use of Laboratory Animals on a protocol approved by the Institutional Animal Care and Use Committee (IACUC). Subcutaneous xenograft studies were performed as previously reported [12]. Five-week-old female athymic nude mice were purchased from Envigo (Indianapolis, IN) or Charles River (Wilmington, MA) and maintained in the institutional animal facilities approved by the American Association for Accreditation of Laboratory Animal Care. Athymic nude mice were injected in the right flank area with human breast cancer, MDA-MB-231 cells (5 × 10^6^) in 100 μL of PBS, and when tumors have reached 80–120 mm^3^ (measured by calipers) animals were grouped so that the mean tumor sizes of each group were nearly identical and tumor-bearing mice were treated with different regimen in separate studies as follows: tumor-bearing mice were administered H182 via oral gavage every day at 5 mg/kg for 19 days and 20 mg/kg for additional 14 days, H182 via intraperitoneal (IP) injection every other day at 10 mg/kg for 33 days, and H120 via oral gavage every day at 10–30 mg/kg for 28 days. Tumor sizes were monitored every 3–4 days, measured with calipers, and converted to tumor volume, *V*, according to the formula *V* = *0.52* × *a*^*2*^ × *b*, where *a*, smallest superficial diameter, and *b*, largest superficial diameter. For each treatment group, the tumor volumes for each set of measurements were statistically analyzed relative to the control (DMSO-treated; 70% DMSO with sterile PBS) group.

For syngeneic models, 6–10-week-old female C57BL/6 mice were purchased from Jackson Labs (Bar Harbor, ME) and used for the experiments. To generate tumors, 1 × 10^6^ E0771 murine breast cancer cells, which are syngeneic to C57BL/6, were injected in the mammary gland [17]. For analysis of the experimental murine data, mice with insufficient or missing data were censored for the analysis. Radiation was delivered to the tumor only using the X-RAD SmART (PXi, Precision X-Ray, North Branford, CT) once tumors reached 800–1000 mm^3^. Mice were anesthetized with isoflurane and then placed inside the shielded cabinet. A computed tomography (CT) scan was then performed to determine the location of the tumor. Once the location was determined on the CT, the treatment beams were set up on the mice using the accompanying planning software package (SmART Advanced Treatment Planning System, Precision X-Ray). Collimated opposed tangential beams with an energy of 225 kV were utilized to treat the tumor and minimize the dose to the surrounding normal tissue. The Stat3 inhibitor, H278 (HCl salt of H182) was prepared and delivered intraperitoneally at a concentration of 10 mg/kg. H278 was diluted in a 70% DMSO with sterile PBS and prepared from a 100 mM DMSO stock. Mice were given H278 at 10 mg/kg intraperitoneally every other day alone and in combination with radiation starting 3 days prior to RT and continuing for the duration of treatment. For tumor growth, all measurements at each time point for a given treatment group were averaged and a standard error was calculated. Comparisons between groups were then done using ANOVA with post-hoc analysis. Survival curves were generated using the IACUC mandated endpoint of when a tumor reached >3500 mm^3^ (or > 20 mm in any one dimension). Mice that reached experimental endpoint (harvested for analysis), but not survival endpoint were censored from that time point forward. Comparisons between groups were then performed using a Log-Rank test. Standard star notations (**p < 0.01) were used to designated level of significance for all comparisons.

## 3. Results

### 3.1. Discovery of azetidine lead compound H182 from medicinal chemistry/SAR-directed optimization of carboxylic acid-based Stat3 inhibitors

**Fig. 1.**
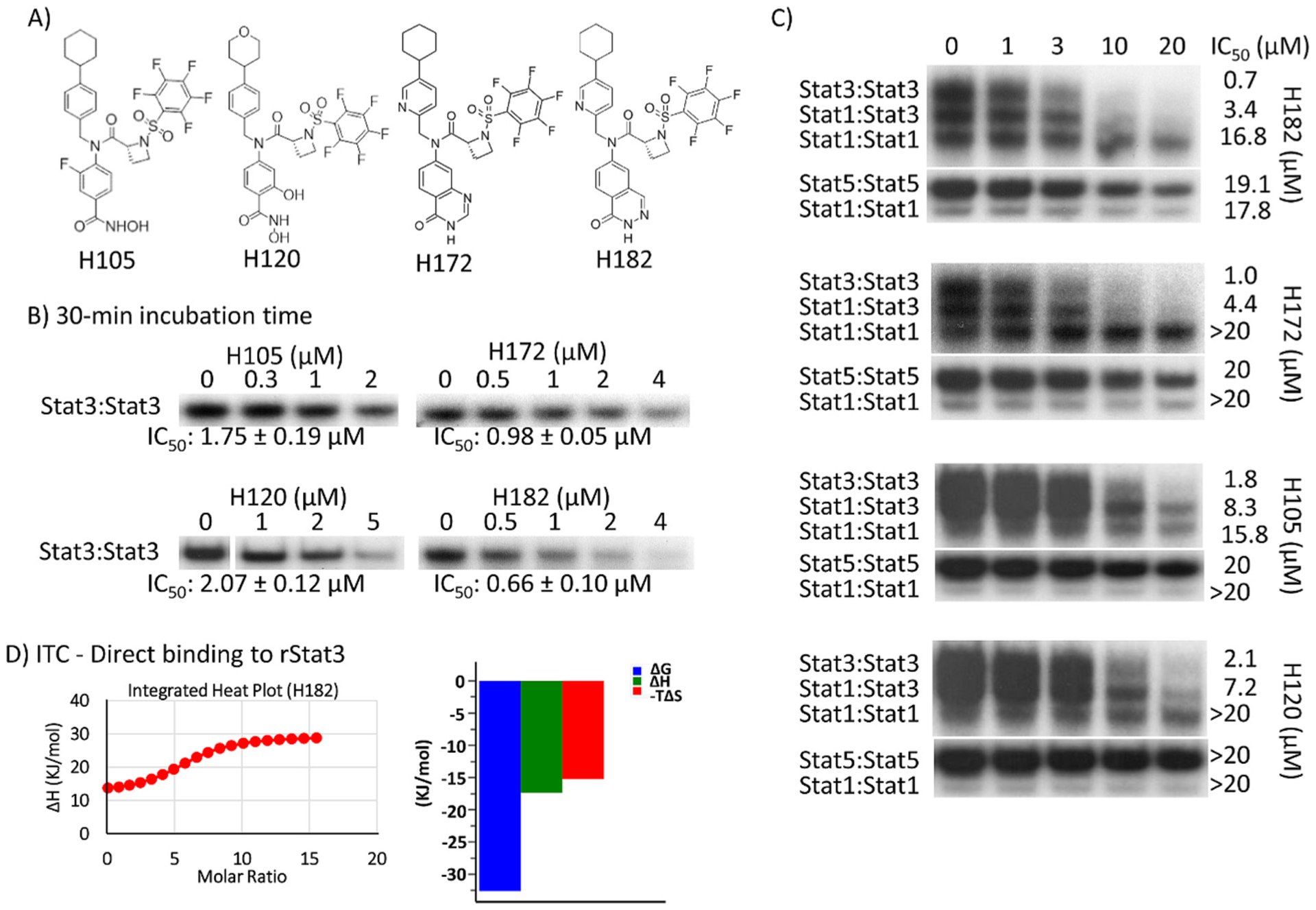
Compounds, H105, H120, H172, and H182, and their effects against *in vitro* Stats DNA-binding activity, and H182 interactions with Stat3. (A) Structures of H105, H120, H182, and H172; (B, C) EMSA analysis of Stats DNA-binding activities in nuclear extracts of equal total protein containing activated (B) Stat3, or (C) Stat1, Stat3, and Stat5 pre-incubated with increasing concentration of the indicated compounds for 30 min at room temperature prior to incubation with the radiolabeled hSIE probe that binds Stat1 and Stat3 or the MGFe probe that binds Stat1 and Stat5 and then subjecting to native gel electrophoresis; and (D) Isothermal titration calorimetry and the binding isotherm from the integrated thermogram fit using the one-site model in the PEAQ-ITC software generated from the titration of H182 into Stat3, and the signature plots of the thermodynamics parameters, ΔG, ΔH, and −TΔS. Positions of Stats:DNA complexes in gel are labeled; control lanes (0) represent nuclear extracts pre-treated with 10% DMSO. Bands corresponding to Stats:DNA complexes were quantified using ImageJ, calculated as percent of control, and plotted against concentration to derive IC 50 values, which are shown. Data are representative of 2–4 independent determinations.

The insight from having worked extensively on the carboxylic acid-based leads, BP-1–102 [12], SH4–54 and SH5–07 [13] led us to design a new series of structurally unique inhibitors, H120, H105, H172, and H182, which contain a key azetidine ring (Fig. 1A). Compounds, H120, H105, and H172 with the nomenclature **8e**, **8f**, and **9f**, and details of how the novel azetidine-based inhibitors were derived are disclosed in our previous articles [14,15]. Additional information on the synthesis and characterization of H182 and H172 is provided in the Supplementary Information “Methods”.

### 3.2. Azetidine functionalized compounds showed higher potency and selectivity against Stat3:Stat3 DNA-binding activity in vitro over that of Stat1 or Stat5 activity

The activities of H105, H120, H172, and H182 against Stat3, Stat1, or Stat5 DNA-binding activity *in vitro* were determined by the standard electrophoretic mobility shift assay (EMSA) [11–13]. In this assay, nuclear extracts of equal total protein containing activated Stat3 prepared from NIH3T3/v-Src fibroblasts, or containing activated Stat1, Stat3 and Stat5 prepared from epidermal growth factor (EGF)-stimulated NIH3-T3/hEGFR fibroblasts were pre-incubated with increasing concentration of the compounds at room temperature for 30 min, prior to incubation with the radiolabeled high-affinity *sis*-inducible element (hSIE) probe that binds Stat3 and Stat1 or the mammary gland factor element (MGFe) probe that binds Stat1 and Stat5 and subjecting to gel shift analysis [10, 12]. Results showed a dose-dependent inhibition of Stat3 DNA-binding activity (Fig. 1B), with IC_50_ values of 0.66 ± 0.10, 0.98 ± 0.05, 1.75 ± 0.19, and 2.07 ± 0.12 μM, respectively, for H182, H172, H120, and H105. The activities of H172 and H182 are much improved over those (IC_50_ values of 4–7 μM) of the previous lead inhibitors, BP-1–102, SH5–07 and SH4–54 [12,13]. These data also showed the preferential inhibition of Stat3:Stat3 DNA-binding activity, ahead of the inhibition of Stat1:Stat3 (IC_50_ of 3.4–8.3 μM), with far lower potencies against Stat1:Stat1 (IC_50_ > 15.8 μM) and Stat5:Stat5 activities (IC_50_ values > 19.1 μM) (Fig. 1C).

To demonstrate that H182 and the azetidine compounds directly bind to Stat3, we performed isothermal titration calorimetry (ITC) studies [20] using purified recombinant Stat3 (rStat3), which contains the core fragment of human Stat3 (amino acids 127 to 711) (Fig. 3A), with a C-terminal 6 × His tag [19]. The binding isotherm from the integrated thermogram fit, using the one-site model in the PEAQ-ITC software, generated from the titration of H182 into rStat3 derived the thermodynamics parameters, ΔH of −17.4 kJ/mol, −TΔS of −15.2 kJ/mol, a ΔG of −32.6 kJ/mol (Fig. 1D), suggesting spontaneity of the binding. The ITC study derived a *K*_D_ of 1.97 μM (Fig. 1D). Together these data indicate that the azetidine-based inhibitors directly bind to Stat3.

### 3.3. Covalent binding mechanism of action and irreversible inhibition of Stat3 activity from SAR analysis, supported by biochemical, mass spectrometry, and mutagenesis studies

**Fig. 2.**
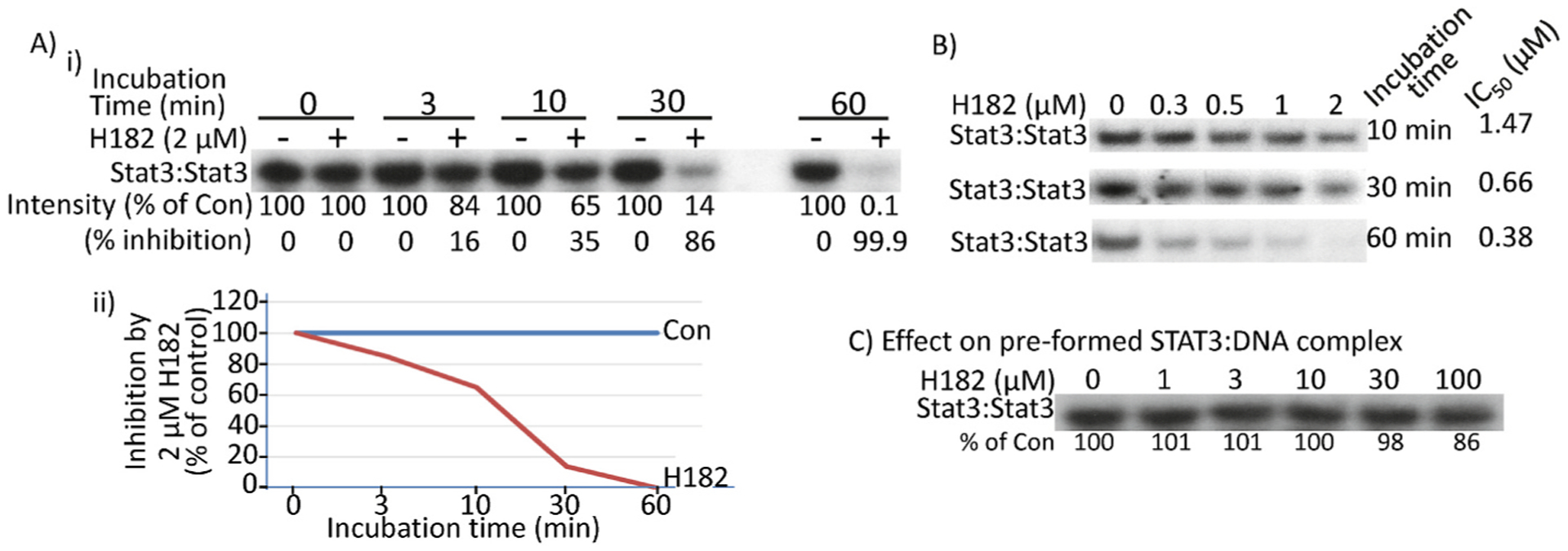
Time-dependency of the inhibition of Stat3 DNA-binding activity by the azetidine compound, H182. (A–C) EMSA analysis of Stat3 DNA-binding activities in nuclear extracts of equal total protein containing activated Stat3 pre-incubated with (A) 2 μM H182 for 3, 10, 30, and 60 min, or (B) 0.3–2 μM H182 for 10, 30, or 60 min at room temperature prior to incubation with the radiolabeled hSIE probe that binds Stat3; or (C) the hSIE probe for 30 min at room temperature prior to incubation with 0–10 μM H182 for 30 min then subjecting to native gel electrophoresis; Positions of Stat3:DNA complexes in gel are labeled; control lanes (0) represent nuclear extracts pre-treated with 10% DMSO. Bands corresponding to Stat3:DNA complexes were quantified using ImageJ, calculated as percent of control (% of con) and shown or plotted against concentration from which IC50 values were derived, which are shown, or the band intensity is plotted against the time of incubation and shown. Data are representative of 2–4 independent determinations.

Substituting the *para*-fluorine of the inhibitor perfluorophenyl-sulfonamide system strongly compromises binding to Stat3 [15]. For example, replacing the *para*-fluorine with Cl or H, as in H186 (**11a**) or H176 (**11b**), respectively led to loss of Stat3-inhibitory potency, while H142 (**5p**) [15], with the *para*-fluorine has strong Stat3-inhibitory activity, IC_50_ of 0.46 μM (Table 1). Results together suggest that the *para*-fluorine is essential to maintain high potency, possibly being displaced through a nucleophilic aromatic substitution (S_N_Ar) mechanism of action [15]. Inhibition through a covalent binding mechanism is time-dependent [21,22]. We hypothesized that the potency of inhibition by the azetidines would be influenced by the duration of Stat3 interaction with the azetidines. To investigate the time-dependency of the inhibition, we used H182 to conduct a time-course EMSA analysis in which nuclear extracts of equal total protein were each incubated with 2 μM H182 for 10, 30, and 60 min, prior to incubation with the labeled hSIE probe and subjecting to gel shift analysis. Results showed a progressively stronger inhibition of Stat3 DNA-binding activity with increasing time of incubation from 10 to 60 min, with a complete inhibition at 60 min (Fig. 2Ai). The plot of the percent inhibition with time is shown in Fig. 2Aii. Dose-response studies of H182 against Stat3 DNA-binding activity for the 10 min and 60 min incubation times derived the IC_50_ values of 1.47 and 0.38 μM, respectively (Fig. 2B), while the IC_50_ value is 0.66 μM for 30 min incubation (Figs. 1B and 2B). We surmise that the longer incubation time allows for robust interactions between the compounds and Stat3, facilitating the covalent interaction of Stat3:compound with time to completion. The course of the inhibition by the azetidines was further probed. In this case, the Stat3 nuclear extracts were pre-incubated with the hSIE probe for 30 min (to promote the DNA-binding event) prior to the incubation with H182 for 30 min and performing EMSA analysis. H182 was unable to inhibit the pre-formed Stat3:DNA complex up to 100 μM (Fig. 2C), indicating that the Stat3 pre-complexation with the high-affinity oligonucleotide interferes with the ability of H182 to effectively engage with Stat3.

**Schemes 1 and 2.**
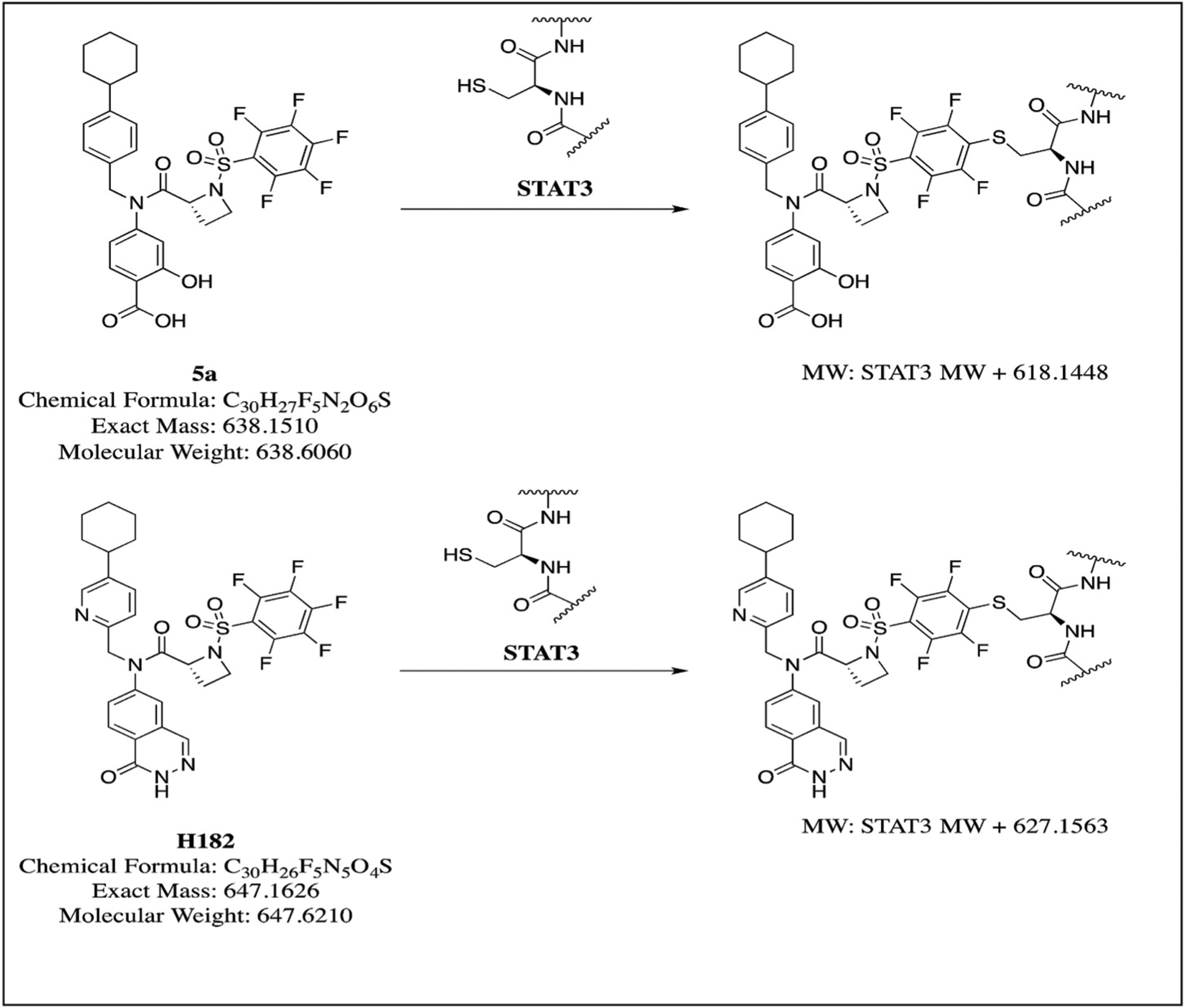
Expected reaction of H098 (**5a**) or H182 with Stat3 Cys residue.

We next employed nano-LC/MS/MS proteomics approach to study whether the azetidine compounds covalently interact with Stat3 protein’s cysteine residues *in vitro* and selected H098 (**5a**) [15] and H182 for these studies. We utilized the same rStat3 protein that was used in the ITC assay, which contains amino acids 127 to 711 and a total of 10 cysteine residues in its amino acid sequence. We needed to detect all these 10 cysteine residues with MS/MS spectra if possible, which could be challenging. Therefore, we used a comprehensive method by way of multiple enzymatic digestions and multiple MS/MS fragmentation techniques in order to maximize Stat3 sequence coverage. Overall, with these exhaustive efforts, we were able to achieve more than 75% sequence coverage with more than 2800 peptide spectrum matches (PSMs) at 5% false discovery rate (FDR), and 8 out of 10 cysteines were detected from these PSMs (data not shown). We hypothesized that if the compounds were able to interact with the sulfhydryl (-SH) group of the Cys residues covalently, each Cys residue will increase its mono-mass by 618.1448 and 627.1563 Da for H098 [15] and H182, respectively (Schemes 1 and 2). We used these mass increases as variable modifications for Cys to perform MASCOT database search and discovered that, indeed, several Cys residues were modified by the two compounds as summarized in Table 2. These experiments were repeated three or two times as indicated with “experimental reproducibility” in the table. The results indicate that compound H098 is able to react with Cys residues C328 and C426, and compound H182 can react with C468 and C542. Therefore, we conclude that compounds H098 and H182 have the ability to covalently interact with Stat3 protein *in vitro*.

**Fig. 3.**
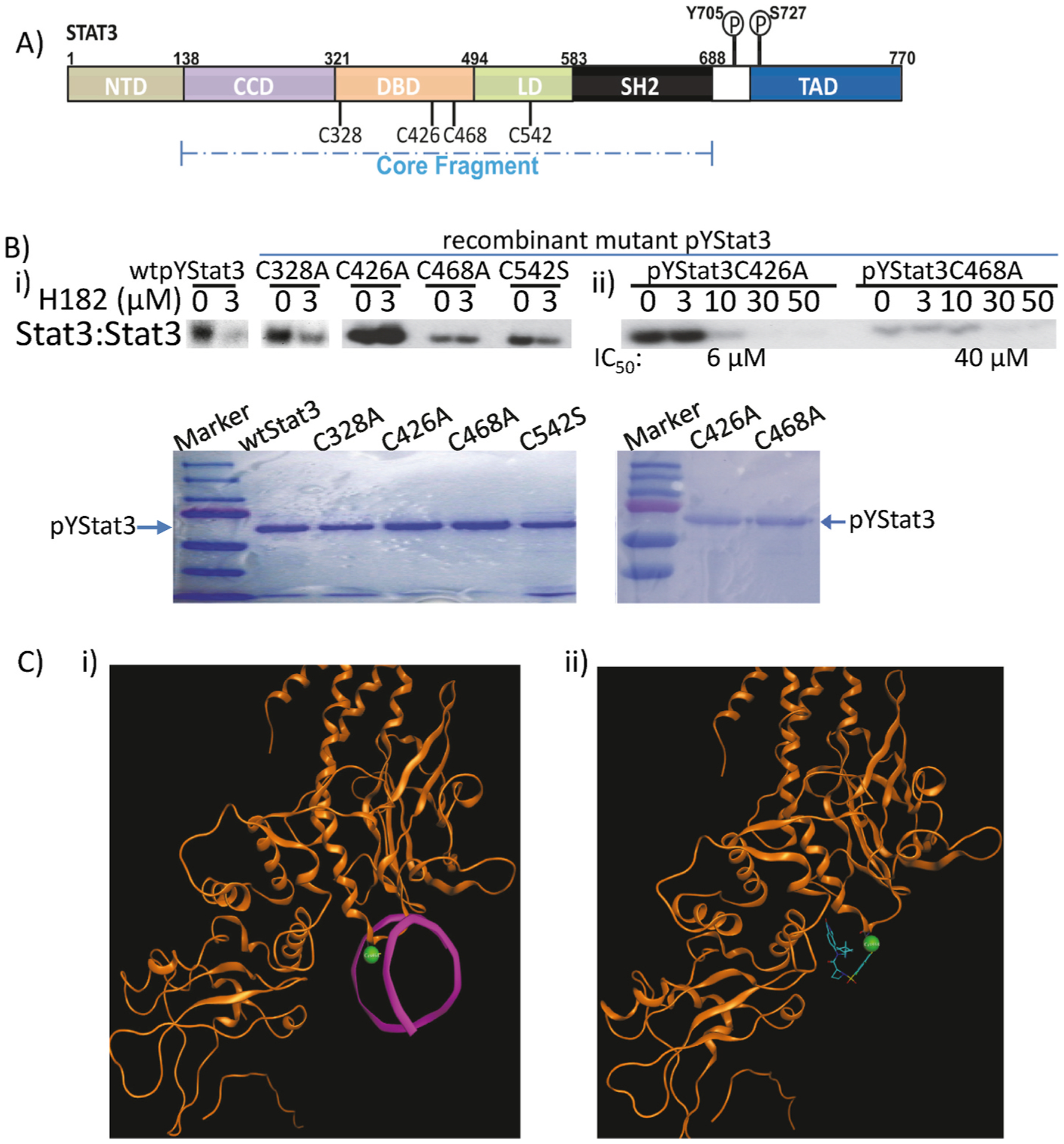
Site-directed mutagenesis identifies cysteine residues in Stat3 that covalently interact with the azetidine inhibitors. (A) Stat3 domain structure and the cysteine residues identified by mass spectrometry to interact with H098 and H182; NTD, N-terminal domain, CCD, coiled-coil domain, DBD, DNA-binding domain, LD, Linker domain, SH2, Src homology-2 domain, and TAD, transcriptional activation domain, P, phosphate; (B) Stat3 DNA-binding activities in lysates of equal total protein containing pure (i) wild-type (wt) pYStat3, or the mutant pYStat3C328A, pYStat3C426A, pYStat3C468A, or pYStat3C542S pre-incubated with 0–3 μM H182, or (ii) pYStat3C426A or pYStat3C468A pre-incubated with 0–50 μM H182 for 30 min at room temperature prior to incubation with the radiolabeled hSIE probe that binds Stat3, and then subjecting to native gel electrophoresis (upper); protein loading shown by SDS-PAGE (lower); and (C) i) Crystal representation of Stat3 monomer bound to DNA (magenta), highlighting the Cys468 residue (green), which is in the binding domain, within 4.2 Å of direct contact with DNA (PDB code 1BG1), and ii) Modeling representation of Stat3 with Cys468 (green) covalently bound to H182 low energy conformation (blue, with nitrogen in dark blue, oxygen in red, sulfur in yellow, and fluorine in green). (Modeling and visualization used MOE software). Positions of Stat3:DNA complexes or Coomassie-stained protein in gel are labeled; control lanes (0) represent extracts pre-treated with 10% DMSO. Bands corresponding to Stat3:DNA complexes were quantified using ImageJ, calculated as percent of control, and plotted against concentration from which IC50 values were derived. Data are representative of 2–3 independent determinations.

Given the MS data, we conducted site-directed mutagenesis analysis for the effects of Cys mutations (Cys-to-Ala or Cys-to-Ser mutations) on the inhibitory activities or potencies of the compounds. We used the same rStat3 construct that contains the core fragment of human Stat3 protein from amino acids 127 to 711 [19], as shown in Fig. 3A and stated in the “Methods” section to generate the Cys mutants. Purified, phosphorylated wild-type (wt)Stat3, and phosphorylated mutants, Stat3C328A, Stat3C426A, Stat3C468A, and Stat3C542S protein samples of equal amounts were pre-incubated with or without 3 μM H182 for 30 min at room temperature prior to incubation with the radiolabeled hSIE probe that binds Stat3 and subjecting to EMSA analysis. Results showed strong inhibition of the DNA-binding activities of wtpYStat3, pYS-tatC328A, and pYStat3C542S by 3 μM H182, while the DNA-binding activities of pYStat3C426A and pYStat3C468A are unaffected (Fig. 3Bi, compare 0 and 3 μM). These results suggested that C426 and C468 might be key for H182 to bind to and inhibit Stat3 activity. Focusing on the C426 and C468, we next conducted dose-response studies for effects of H182 on Stat3C426A and Stat3C468A. EMSA analysis showed that 10 μM H182 induced near complete inhibition of pYStat3C426A activity, but had no effect on pYStat3C468A activity, which was instead inhibited by 30 μM H182, with IC_50_ values of 6 μM (pYStat3C426A) and 40 μM (pYStat3C468A), with respect to the inhibition by H182 (Fig. 3Bii). Equal proteins used in the DNA-binding assay/EMSA analysis are shown by SDS-PAGE Coomassie staining (Fig. 3B, lower). Thus, we conclude that though both C426 and C468 are contributing to the binding of Stat3 with the azetidine compounds, the results suggest the C468 represents the predominant residue driving the interactions with the H182. Ribbon diagram of Stat3 monomer bound to DNA (PDB code 1BG1) highlighting Cys468, which is within 4.2 Å of direct contact with DNA as generated by Molecular Operating Environment (MOE) software (MOE 2020.09, Chemical Computing Group, Montreal, Quebec, Canada (2021)) [23] are shown in Fig. 3C.

The Stat3:azetidine compound interaction was investigated further by focusing on how the compounds might interfere with Stat3 dimerization (Stat3:Stat3), which occurs via pTyr peptide:SH2 domain interaction. We employed the Stat3 fluorescence polarization (FP) assay [11, 12,24], which is designed to model the Stat3:Stat3 dimerization [25,26] and uses 5-carboxy fluorescein-labeled high affinity pTyr peptide, GpYLPQTV-NH_2_ to bind to the SH2 domain of pure recombinant Stat3 (5-fl-GpYLPQTV-NH_2_:Stat3 complex). The GpYPQTV-NH_2_ peptide is derived from the interleukin-6 receptor (IL-6R)/gp130 [24,27] and binds with high affinity (*K*_D_ = 150 nM) to the SH2 domain [27]. As expected, in the FP assay, the presence of 0–10 μM of the unlabeled GpYLPQTV-NH_2_ peptide (at 30 min incubation with Stat3) led to a classical dose-dependent reduction of the FP signal down to the baseline of 45–50 mP, whether the FP signal measurements were taken at 10, 30, or 60 min after adding probe (Supplementary Fig. S1, GpYLPQTV-NH_2_), indicative of the disruption of pTyr:SH2 domain interaction [11,26]. By contrast, the presence of 0–10 μM H182 (at 10–60-min incubation with Stat3) led to fluctuations of the FP signal that are of a different pattern from the dose-dependent reduction of the FP signal observed for the unlabeled GpYLPQTV-NH_2_ peptide (Supplementary Fig. S1, H182), despite the high inhibitory potency (Fig. 1B). Altogether, the Stat3 DNA-binding/EMSA analysis, ITC, and FP results indicate that the azetidine compounds directly bind to Stat3 in a unique and distinct way from SH2 domain-binding molecules.

### 3.4. Azetidine compounds inhibit constitutive and ligand-induced Stat3 activation and block Stat3 nuclear accumulation with no change in Stat3 protein levels in breast cancer cells

**Fig. 4.**
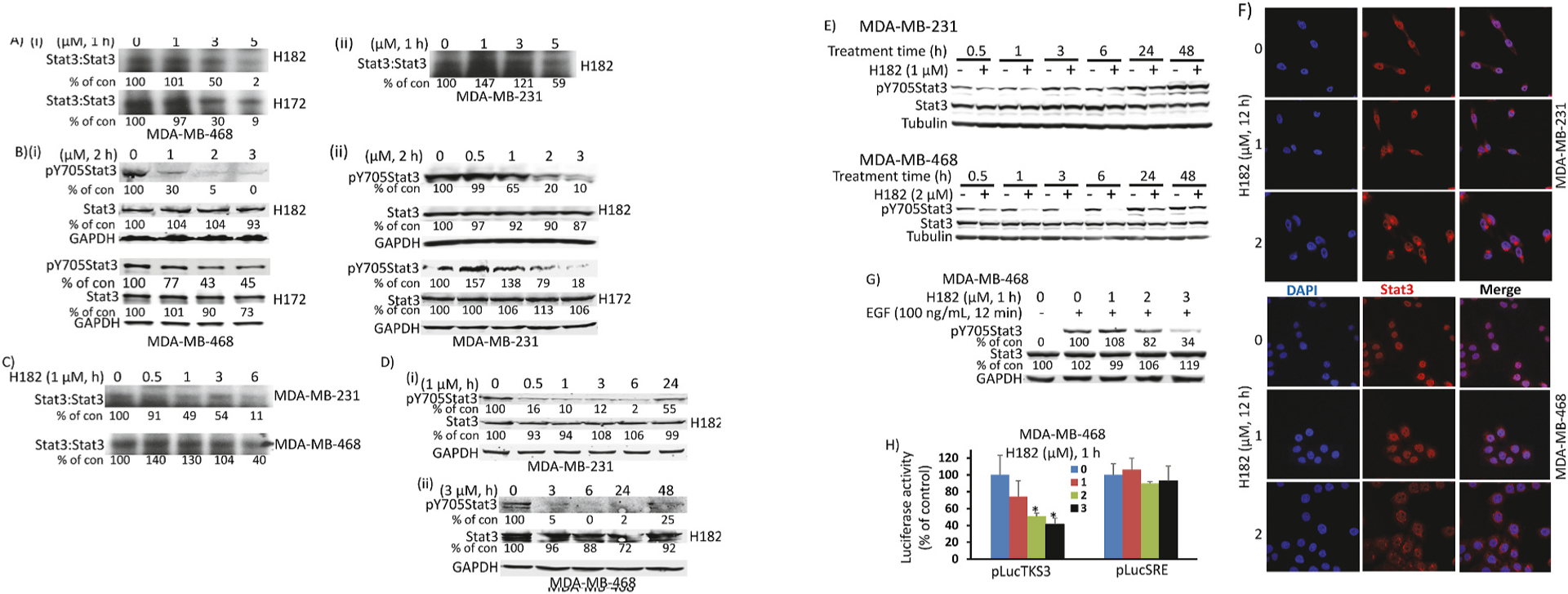
Effects of azetidine-based compounds on the induction of Stat3 phosphorylation, DNA-binding and transcriptional activities in breast cancer cells. (A, C) Nuclear extracts of equal total protein prepared from the indicated breast cancer lines untreated (DMSO, 0) or treated with H172 or H182 at 1–5 μM for 1 h (A), or 1 μM for 0.5–6 h (C) and subjected to EMSA analysis of Stat3 DNA-binding activity using the radiolabeled hSIE probe that binds Stat3; (B, D, E, G) SDS-PAGE and immunoblotting analysis of whole-cell lysates of equal total protein prepared from the indicated human breast cancer lines untreated (DMSO, 0, -) or (B) treated with 0.5–3 μM H172 or H182 for 2 h, (D) treated with 1 or 3 μM H182 for 0.5–48 h, (E) treated with 1 or 2 μM H182 for 0.5–48 h; or (G) serum-starved for 24 h and pre-treated with 1–3 μM H182 for 1 h prior to stimulation with 100 ng/ml EGF for 12 min, and probing for pY705Stat3, Stat3, tubulin, or GAPDH; (F) Immunofluorescence staining with laser-scanning confocal microscopy analysis of MDA-MB-231 and MDA-MB-468 lines untreated (0) or treated with 1–2 μM H182 for 12 h, stained for DAPI (blue) and Stat3 (red). Images were captured for DAPI, Stat3, and merged; and (H) Luciferase reporter activity in the cytosolic extracts of equal total protein prepared from the v-Src-transformed mouse fibroblasts, NIH3T3/v-Src transiently transfected with the Stat3-dependent luciferase reporter, pLucTKS3 or Stat3-independent luciferase reporter, pLucSRE, untreated (DMSO, 0) or treated with 1–3 μM H182 for 1 h. Luciferase reporter activity was assayed with a luminometer, normalized to protein concentration, and plotted as percent of control. Positions of Stat3:DNA complex or proteins in gel are labeled; control lane (0, -) represents nuclear extracts, whole-cell lysates, or cytosolic extracts prepared from 0.5% DMSO-treated cells. Values, mean ± S.D., n = 3, *p < 0.05. Bands corresponding to Stat3:DNA complexes or proteins in gel were quantified using ImageJ, calculated as percent of control, and shown. Data are representative of 2–4 independent determinations.

The breast cancer lines, MDA-MB-468 and MDA-MB-231, which harbor constitutively-active Stat3 [3,13], were used in both dose-response (treated with 0–5 μM H182 or H172 for 1 or 2 h) and time-course studies (treated with 1 or 3 μM H182 and H172, or 5 μM H120 and H105 for 0–24 h) to evaluate the effects of the inhibitors on Stat3 activation. Nuclear extracts and whole-cell lysates were prepared, and samples of equal total protein were subjected to Stat3 DNA-binding assay/EMSA analysis using the hSIE probe, or to SDS/PAGE and Western blotting analysis for pY705Stat3, Stat3, or GAPDH [11,12]. H182 and H172 treatment led to dose-dependent inhibition of constitutive Stat3 DNA-binding activity (Fig. 4A, Supplementary Fig. S2B) and Tyr phosphorylation (Fig. 4B, Supplementary Fig. S2A, pYStat3). Depending on the concentration (1, 3, or 5 μM), the inhibitory effects of the azetidine compounds were observed relatively early, at 30–60 min, on both Stat3 DNA-binding activity (Fig. 4C) and Tyr phosphorylation (Fig. 4D, pY705Stat3). Treatment of cells with the higher, 5 μM concentration leads to an even earlier inhibition of Stat3 activity, as early as 8–15 min (Supplementary Fig. S2A, H120). For the treatment with 1 μM H182, or with the relatively weaker H105, we note that there was a rebound of the pY705Stat3 levels at later time points (3–24 h) (Fig. 4Di, Supplementary Fig. S2A, H105), which was not the case when the cells were treated with the higher concentration 3 μM H182 that caused a sustained inhibition of Stat3 activation for up to 48 h (Fig. 4Dii).

During confluence of cultured cells, the cell-to-cell adhesion and cadherin engagement trigger increased Tyr705Stat3 phosphorylation in a variety of cell lines [28,29]. To investigate the impact of cell-to-cell adhesion and cadherin engagement, parallel studies were conducted in which cells in culture were untreated or treated with 1 or 2 μM H182 for 0.5–48 h, and whole-cell lysates prepared and immunoprobed for pTyr705Stat3. Results showed that with longer duration of culture the pTyr705Stat3 levels increased (Fig. 4E), and that comparing the untreated versus the treated side-by-side, the treatment with H182 led to inhibition of pTyr705Stat3 (Fig. 4E). The inhibition of pTyr705Stat3 at 48-h treatment with H182 was minimal at the concentration of 2 μM and undetectable at 1 μM (Fig. 4E). After 48-h incubation, the cell confluency of the DMSO-treated samples reached to around 60% for MDA-MB-468 cells and 80% for MDA-MB-231 cells. These results indicate that cell-to-cell adhesion and cadherin engagement are part of the mechanism for the re-bounce in pTyr705Stat3 levels following prolonged treatment. We next determined the effects of the azetidine inhibitors on the intracellular localization of Stat3 by performing immunofluorescence staining with laser-scanning confocal microscopy analysis on both MDA-MB-231 and MDA-MB-468 lines treated with H182 for 12 h. In the untreated cells, Stat3 (red punctate) staining was predominantly localized in the nucleus of the tumor cells, with weak presence in the cytoplasm (Fig. 4F, 0, Stat3, red merged with blue DAPI nuclear staining). Treatment with 1 or 2 μM H182 led to decreased nuclear Stat3 staining and Stat3 aggregation at the perinuclear region (Fig. 4F, H182, Stat3, red merged with blue DAPI nuclear staining). These results indicate that the azetidine compounds disrupt Stat3 nuclear accumulation and promote the aggregation of Stat3 at the perinuclear region, consistent with our previous studies [18]. Treatment with 1–3 μM H182 further inhibited EGF-induced pY705Stat3 in MDA-MB-468 cells in a dose-dependent manner (Fig. 4G). Total Stat3 protein levels in all of these studies were unchanged, and GAPDH, tubulin or β-actin levels show equal protein loading. The percent changes induced by H182 treatment on Stat3 DNA-binding activity and phosphorylation in the treated cells are shown.

We next evaluated the effects of the azetidine compound, H182 on Stat3 transcriptional activity. We used mouse fibroblasts transformed by the viral Src oncoprotein (NIH3T3/vSrc) that harbor constitutive Stat3 activation, which were transiently transfected with the Stat3-dependent luciferase reporter, pLucTKS3 [25,30]. Transfected cells were treated with H182 and the luciferase activity was assayed in the cytosolic extract preparations of equal total protein [12,13,30]. Results showed dose-dependent inhibition of the Stat3-driven luciferase reporter expression by the treatment of cells with 1–3 μM H182 for 1 h (Fig. 4H, pLuckTKS3), with significant (*p* < 0.05) decreases at 2 and 3 μM treatments. By contrast, the luciferase reporter assay of the cytosolic extracts from NIH3T3/vSrc fibroblasts transiently transfected with the Stat3-independent pLucSRE luciferase reporter [25,30] showed no changes in the expression of the reporter following similar treatments with H182 (Fig. 4H, pLucSRE).

### 3.5. Azetidine compounds have little or no effects on pS727Stat3, Stat1, JAK2, EGFR, Shc, Erk1/2, Src, or Akt induction in breast cancer cells

**Fig. 5.**
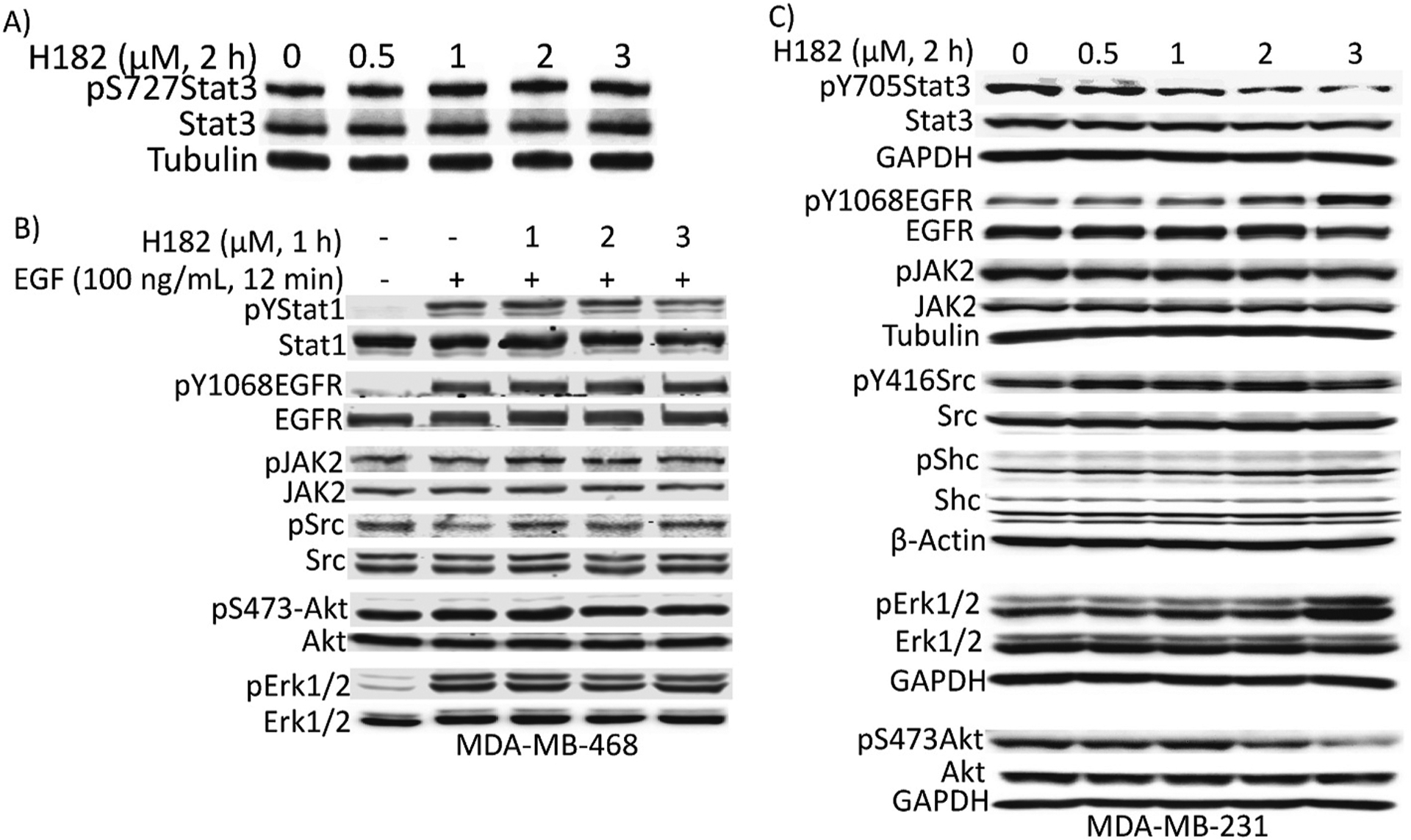
Lack of non-specific effects on other signaling pathways in breast cancer cells. SDS-PAGE and immunoblotting analysis of whole-cell lysates of equal total protein prepared from the indicated human breast cancer lines untreated (DMSO, 0, -) or treated with (A, C) 0.5–3 μM H182 for 2 h, or (B) 1–3 μM H182 for 1 h prior to stimulation with 100 ng/ml EGF for 12 min, and probing for pY705Stat3, pS727Stat3, Stat3, pYStat1, Stat1, pY1068EGFR, EGFR, pJAK2, JAK2, pY416Src, Src, pShc, Shc, pErk1/2, Erk1/2, pS473Akt, Akt, or GAPDH, tubulin, and β-Actin. Positions of proteins in gel are labeled; control lane (0, -) represents whole-cell lysates prepared from 0.5% DMSO-treated cells. Data are representative of 3–4 independent determinations.

We sought to assess any non-specific effects of the azetidine compounds. Breast cancer MDA-MB-231 and MDA-MB-468 lines were treated with H182 for 2 h at concentrations of 0–3 μM that inhibit constitutive and ligand-induced Stat3 activation, stimulated or not with EGF for 12 min, and whole-cell lysates were prepared. Samples of equal total protein were subjected to SDS-PAGE and Western blotting analysis. Treatment with 0.5–3 μM H182 for 2 h had no inhibitory effect on the constitutive or EGF-induced pS727Stat3, pYStat1, pY416Src, pY1068EGFR, pShc, pErk1/2^MAPK^, pS473Akt, and pJAK2 in TNBC cells (Fig. 5A–C), while pY705Stat3 was inhibited (Fig. 5C pYStat3) in the TNBC cells. The total protein levels were unchanged, and the tubulin, β-actin, and GAPDH levels show equal protein loading.

### 3.6. Compounds inhibited anchorage-dependent and independent growth, induced apoptosis, and suppressed Stat3 target gene expression in TNBC cells harboring constitutively-active Stat3

**Fig. 6.**
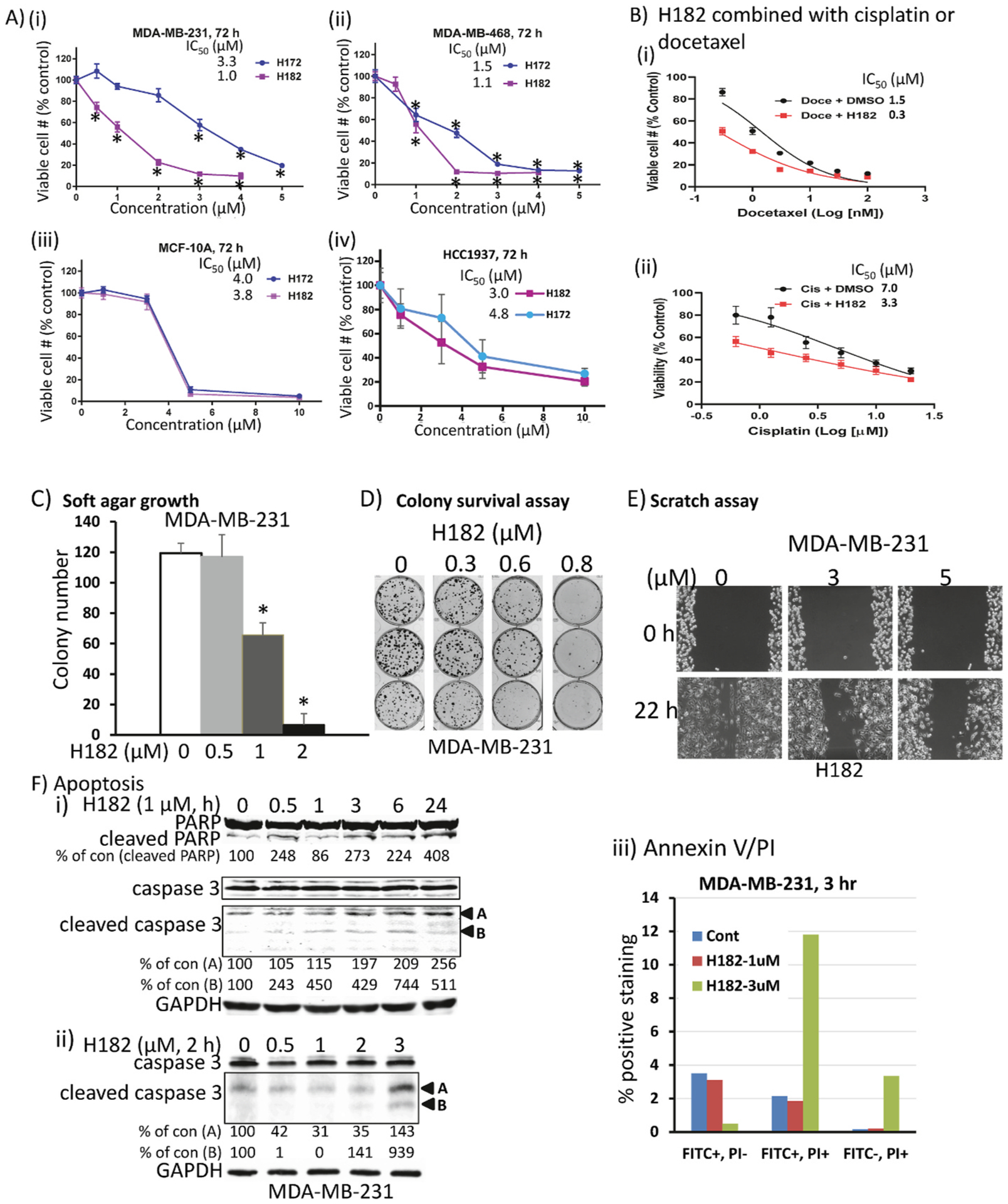
Azetidine-based compounds suppress anchorage-dependent and independent growth, colony survival, and migration, and induce apoptosis of breast cancer cells harboring aberrantly-active Stat3. (A) Human breast camcer, MDA-MB-231 and MDA-MB-468 cells (i and ii) that harbor constitutively-active Stat3 or normal human breast epithelial, MCF-10A or breast cancer HCC1937 cells (iii and iv) that do not in 96-well culture were treated once with increasing concentration of H172 or H182 for 72 h, or (B) MDA-MB-231 cells in 96-well culture, untreated (DMSO) or pre-treated with 1 μM H182 for 6 h prior to treating with 0–100 nM docetaxel (i) or 0–20 μM cisplatin (ii) for a total of 72 h, and viable cell numbers were assayed using CyQuant cell proliferation kit and plotted as % viable cell numbers against concentration, from which IC50 values were derived; (C–E) MDA-MB-231 cells seeded (C) in soft-agar were treated every 3–4 days with 0–2 μM H182 and allowed to grow until large colonies were visible, which were stained with crystal violet, counted and plotted, (D) as single-cell culture and treated once with 0–0.8 μM H182 and allowed to culture until large colonies were visible, which were stained with crystal violet and imaged, or (E) in culture and wounded, and treated once with 0–5 μM H182, and allowed to migrate to the denuded area over a 22-h period and imaged; and (F) MDA-MB-231 cells in culture treated once with H182 at (i) 1 μM for 0–24 h or (ii) 0–3 μM for 2 h, and whole-cell lysates prepared and subjected to SDS-PAGE and immunoblotting analysis for PARP, cleaved PARP, caspase 3, cleaved caspase 3, and GAPDH, or (iii) 0–3 μM for 3 h, and samples prepared and processed for Annexin V binding and flow cytometry. Positions of proteins in gel are labeled; control (0) represents samples from 0.5% DMSO-treated cells. Values, mean ± S.D., n = 3–6, *p < 0.01. Data are representative of 3 independent determinations.

Aberrantly-active Stat3 promotes tumor cell growth and proliferation, survival, migration, invasion, and metastasis [2,3]. We investigated the antitumor effects of the azetidine analogs by treating human breast cancer MDA-MB-231 and MDA-MB-468 lines harboring aberrantly-active Stat3 with increasing concentrations of H105, H120, H172, or H182 for 72 h and performing CyQuant assay for viable cell numbers [13]. All four compounds dose-dependently reduced viable cell numbers (Fig. 6Ai and ii, Supplementary Fig. S3B). H182 had the highest potency against both lines, with an IC_50_ of 1 μM (Fig. 6Ai and ii), H172 was 2-fold more potent against MDA-MB-468 (IC_50_ of 1.5 μM) than against MDA-MB-231 (IC_50_ of 3.3 μM) (Fig. 6Ai and ii), while H120 and H105 had IC_50_ range of 2.6–3.6 μM (Supplementary Fig. S3B). The combined treatment with 1 μM H182 and increasing doses of docetaxel [31] or cisplatin [32] led to a shift of the chemotherapeutic agents’ dose-response curves to the left (Fig. 6B), indicating enhanced responses to the chemotherapeutic drugs when used in combination with H182. The IC_50_ values improved from 1.5 to 0.3 μM for docetaxel alone and docetaxel with H182, respectively, and from 7.0 to 3.2 μM for cisplatin alone and cisplatin with H182, respectively (Fig. 6B). These results are consistent with the role of aberrantly-active Stat3 in suppressing drug sensitivity and promoting drug resistance [33], and together suggest that the azetidine compounds may be used in combination with chemotherapy to enhance therapeutic response. By contrast, treatments with the azetidine compounds showed relatively weaker effects on the viable cell numbers of cells that do not harbor aberrantly-active Stat3. The potencies (IC_50_s) of H172 and H182 are 3.8–4.0 μM on normal human breast epithelial MCF-10A cells, 3.6–4.3 μM on breast cancer HCC1937 cells (Fig. 6Aiii, iv), and 4.9–9.2 μM on normal human mesothelial cells (Supplementary Fig. S3A), while the IC_50_s of H120 and H105 on MCF-7 or MCF-10A cells are 7.7 - >10 μM (Supplementary Fig. S3C). Furthermore, treatment of MDA-MB-231 cells with 0.3–2 μM H182 potently and dose-dependently inhibited both anchorage-independent growth in soft-agar (Fig. 6C), and anchorage-dependent colony formation (Fig. 6D). In a scratch assay, one-time treatment of MDA-MB-231 cells with H182 inhibited the cell migration into the denuded area within 22 h of treatment (Fig. 6E). Immunoblotting analysis of whole-cell lysates from MDA-MB-231 cells treated with H182, single concentration (1 μM) for 0–24 h or increasing concentration for 2 h showed moderate levels of cleaved poly ADP-ribose polymerase (PARP) and caspase 3 (Fig. 6Fi and ii), while annexin V/propidium iodide (PI) staining with flow cytometric analysis showed roughly 12% of double-positive staining for cells treated with 3 μM H182 for 3 h (Fig. 6Fiii; Supplementary Fig. S3D, H182, 3 μM) indicative of late apoptosis. Moreover, consistent with constitutively-active Stat3’s role in dysregulating gene expression [1–3], immunoblotting analysis showed that the expression of classical Stat3 downstream genes, including c-Myc, survivin and vascular endothelial growth factor (VEGF), was rapidly suppressed in MDA-MB-231 cells in response to H182 treatment at doses that inhibit Stat3 activity (Supplementary Fig. S4). Together these studies show that the azetidine compounds inhibit aberrantly-active Stat3 signaling and functions in TNBC cells and induce antitumor cell effects *in vitro*.

### 3.7. H120 and H182 inhibited growth of breast tumors in mice, enhanced response to radiation and prolonged survival

**Fig. 7.**
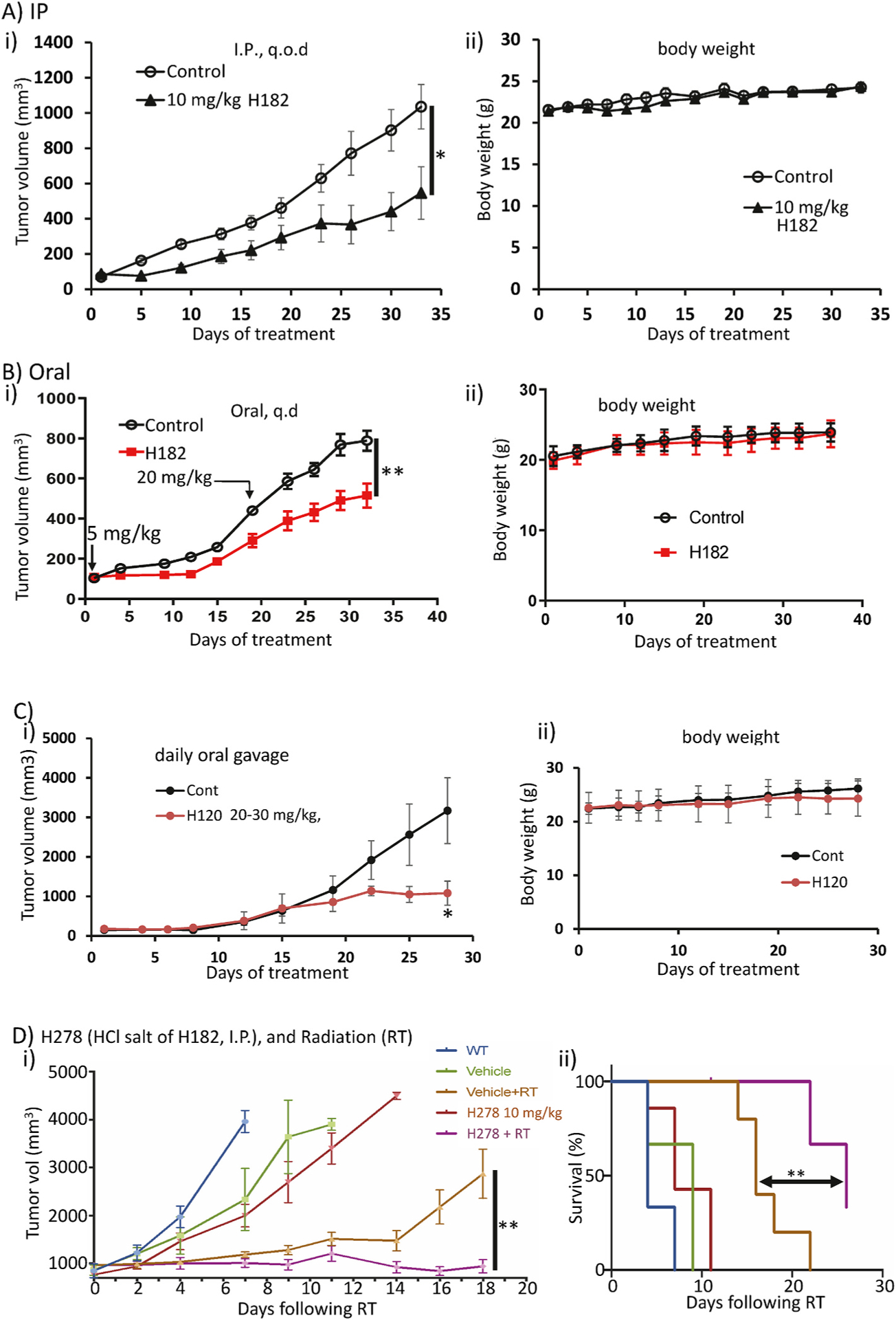
*In vivo* antitumor efficacy against the growth of breast cancer xenografts and syngeneic tumors in mice. (A–C) Plots of tumor growth (i) and body weights (ii) of mice bearing subcutaneous human breast, MDA-MB-231 tumor xenografts. Anti-tumor effects of A) H182 administered via i.p., 10 mg/kg, every 2 or 3 days (*p < 0.05; values, mean ± S.D., n = 5; vehicle, 70/30, DMSO/normal saline v/v), B) H182 administered via oral gavage, 5 mg/kg, every day for 20 days, followed by 20 mg/kg every day for 12 days (**p < 0.01; values, mean ± SEM., n = 5; vehicle, 60/40, DMSO/normal saline v/v), or C) H120 administered via oral gavage, 20 mg/kg, every day for 14 days, followed by 30 mg/kg every day for 14 days (*p < 0.05; values, mean ± S.D., n = 5; vehicle, 5/95, DMSO/sesame oil v/v), compared to vehicle alone (control); and D) mouse bearing orthotopic E0771 mammary tumors were treated with H278, 10 mg/kg, via i.p., every other day, and after one week, a localized kV irradiation (16 Gy) was added or not, and the H278 treatment was continued for the duration of study. Mean tumor burden ± SEM (i) and survival (ii) for the indicated treatments were assessed every 3 days until endpoint (**p < 0.01, mean ± S.D., n = 3 (WT), 3 (vehicle), 5 (vehicle + RT), 7 (H278), and 7 (H278 + RT), vehicle alone (control, 70/30, DMSO/normal saline v/v).

Constitutively-active Stat3 promotes primary tumor development and the progression to therapy resistance [3]. We proceeded to evaluate the *in vivo* antitumor efficacy against tumor growth in both human xenograft and mouse syngeneic models of TNBC. In four separate studies, we tested the efficacy of H182, H278 (HCl salt of H182), and H120. For these xenografts, the human breast cancer, MDA-MB-231 cells were injected subcutaneously into athymic nude mice. When tumors were palpable (80–120 mm^3^), mice were grouped so that the mean tumor sizes were identical between groups and treated as follows: A) intraperitoneal (IP) injections of H182 at 10 mg/kg every other day for 33 days; B) oral gavage of H182 every day at 5 mg/kg for 20 days, and then increased to 20 mg/kg for additional 12 days; and C) oral gavage of H120 at 20 mg/kg, daily for 14 days, and then increased to 30 mg/kg, daily for additional 14 days. These doses were chosen based in part on our previous studies with the earlier lead inhibitors [12,34]. In all three independent studies, significant tumor growth inhibition was observed for H182 and H120 (Fig. 7Ai–Ci). No notable changes in body weights (Fig 7Aii-Cii) or obvious signs of toxicity, such as anorexia, lethargy or change in body condition score were observed.

Given that Stat3 activity promotes chemotherapy resistance [35,36], and the results that H182 in combination with cisplatin or docetaxel had a synergistic effect against breast cancer cells *in vitro* (Fig. 6B), we were interested to determine the antitumor efficacy of combining H182 and radiation therapy. Syngeneic E0771 murine breast cancer cells were injected into the mammary gland of C57BL/6 mice [17], and when tumors were palpable (800–1000 mm^3^), mice were grouped so that the mean tumor sizes were identical between groups. Treatment was initiated as follows: wt (no treatment), vehicle, vehicle and radiation (16 Gy) delivered focally to the tumor using the X-RAD SmART (PXi, Precision X-Ray, North Branford, CT), H278 (HCl salt of H182, 10 mg/kg, I.P., every other day) alone, and H278 (10 mg/kg, I.P., every other day) together with radiation (16 Gy). Both tumor growth and survival were monitored and showed that while H278 alone did not have significant effect, the combination of H278 and focal radiation completely inhibited tumor growth and improved survival in this mouse model of breast cancer (Fig. 7D).

We then profiled tumor tissues for Stat3 activity and Stat3 target gene expression as pharmacodynamic markers of therapeutic response. Tissue lysates of equal total protein prepared from the extracted tumors were subjected to Stat3 DNA-binding assay/EMSA analysis or SDS-PAGE and immunoblotting analysis. Results showed that H182 treatment suppressed Stat3 DNA-binding activity in tumor tissues (Supplementary Fig. S5A, treated T vs. untreated control, C), in parallel with the suppression of the expression of Stat3 downstream genes, including c-Myc, VEGF, and survivin (Supplementary Figs. S5B and T), compared to untreated control tumors. These results validate the inhibition of Stat3 activity and together indicate that the inhibition of constitutively-active Stat3 functions leads to the suppression of growth of TNBC in mice and sensitizes tumors to radiation.

### 3.8. Initial evaluation of DMPK parameters of the azetidine compounds

The *in vivo* activity of a compound is influenced by its overall bioavailability, which is also driven by parameters, such as solubility, permeability, and metabolism [37,38]. In order to assess the physicochemical properties of the azetidine compounds, we conducted the industry standard evaluation of H182 and H172 for solubility, metabolism, CYP inhibition, plasma protein binding, plasma stability, and CEREP SafetyScreen44^™^ [39] via the contract clinical research organization (CRO), Eurofins-CEREP. The results of these studies showed that H172 has aqueous solubility of 104 and 123 μg/ml, while H182 has solubility of 130 and 128 μg/ml, respectively, for simulated gastric fluid (SGF) and simulated intestinal fluid (SIF) (Table 3). These values are all above the industry cut-off of 60 μg/ml [40]. However, H172 and H182 have low solubility of 6.3 and 3.0 μg/ml, respectively, in PBS (Table 3). For metabolic stability, H182 has half-life >150 and 21 min for mouse liver microsome (MLM) and human liver microsome (HLM) assays, respectively, both of which are above the industry cut-off of HLM t _½_ >15 min [41,42]. It has CL_int_ in human hepatocytes of 16 μL/min/10^6^ cells, which falls into moderate stability range [43]. Metabolic stability for H172 was not determined.

For plasma stability, H182 shows a decay from 100% to 53% and 69%, respectively, in human and mouse plasma after 1 h, and to 10% and 23%, respectively, after 2 h (Table 3). H172 was not evaluated. Moreover, H182 plasma protein binding is over 99% for both human and mouse, and it has cytochrome (CYP) inhibition profiles as follows: CYP3A, IC_50_ 6.9 and 1.2 μM at midazolam and testosterone sites, respectively; and the CYP2C9 or CYP2D6 inhibition by 10 μM H182 is 33% or 9%, respectively. Further, the CEREP SafetyScreen44^™^ assay showed only the hERG (human Ether-à-go-go-Related Gene), glucocorticoid receptor (GR), and norepinephrine transporter (NET) had over 80% inhibition at 10 μM H182. We thus conducted dose-response studies for IC_50_, which gave hERG IC_50_ > 10 μM (Qpatch assay), GR IC_50_ 0.11 μM, and NET IC_50_ 0.40 μM (Table 3). These results together suggest the azetidine compounds overall have good properties, though we can optimize the drug metabolism and pharmacokinetic (DMPK) properties further to make the antitumor efficacy even stronger.

## 4. Discussion

Described herein are four members of the new class of azetidine-based small molecules [15], H105, H120, H172, and H182, which directly inhibit Stat3 activity, with a range of potencies in the nanomolar to low micromolar. While many Stat3 inhibitors have been reported [10, 13,14,18,44–48], the *in vitro* potencies (IC_50_s) of 0.38–0.98 μM make H172 and H182 two of the most potent, direct Stat3 inhibitors. The conformational constraints engendered by the azetidine ring led to stronger interactions with Stat3, which result in enhanced potency. The observation that replacing the perfluorobenzene-sulfonamide *para-*f-fluorine atom with any other substituent compromises the Stat3-inhibitory potency [15], suggests that the azetidine-based compounds engage in covalent interactions with Stat3. This hypothesis is supported by the time-dependency of the potency of covalent inhibitors [49], with the potency of Stat3 inhibition by H182 improving from 1.47 μM to 0.66 μM–0.38 μM for 10 min, 30 min, and 60 min incubation with Stat3, respectively. Nano-LC/MS/MS proteomics approach shows that the previously reported azetidine compound H098 reacted with C328 and C426, and the compound H182 reacted with C468 and C542, indicating that the azetidine compounds are capable to covalently interact with Stat3 protein *in vitro*. These findings were confirmed by site-directed mutagenesis that showed the Cys426 and Cys468 residues to be essential for the high potency, though based on the site-directed mutagenesis, Cys468 alone appears to be sufficient to impact the binding to the compounds (Fig. 3Cii). The higher potency with incubation time suggests the irreversible Stat3:H182 adduct formation leads to the exhaustion of the target (Stat3) [49]. The recent successes with the allosteric irreversible inhibitors of mutant KRas that are in clinical trials highlight the value of covalent inhibitors as an approach to effectively inhibit otherwise challenging and intractable targets [50,51].

The cellular activities of the new azetidine analogs are similarly improved over the previous Stat3 inhibitors [3]. H182 shows cellular activities at 1 μM. By interacting with Stat3, the azetidine compounds inhibits constitutively-active ligand-induced Stat3 phosphorylation, DNA-binding activity, and transcriptional activity in human TNBC cells. The compounds further inhibited Stat3 nuclear accumulation and promoted Stat3 perinuclear aggregation in the tumor cells. By contrast, the lack of inhibitory responses against pYStat1, pS727Stat3, pYStat5, JAK2, Src, Shc, pEGFR, Erk1/2 and Akt by the azetidine compounds at concentrations that inhibit Stat3 activity is indicative of the minimal off-target effects. This is consistent with the overall selectivity profile of H182, as determined also from the CEREP SafetyScreen44^™^ *in vitro* panel, which showed that apart from GR and NET receptors (IC_50_s 0.11 and 0.40 μM, respectively), data on hERG inhibition and on the affinities for a number of other receptors, including GPCR, ion channels, transporters, neurotransmitters, NHR, and kinases (data not shown) indicated good off-target properties. This correlates with the *in vivo* studies that showed little evidence of toxicity in mice.

In concordance with the *in vitro* activities, H182 and H120 formulations for oral or intraperitoneal administrations are efficacious against human TNBC xenografts as a standalone, and H278 (HCl salt of H182) in combination with radiation therapy completely blocked tumor growth and prolonged survival in mouse TNBC in syngeneic models. The Stat3-dependency of the antitumor effects is supported by the suppression of the Stat3 target genes, c-Myc, VEGF, and survivin *in vitro* and *in vivo* in response to treatment with H182. Physicochemical property limitations are some of the factors that have precluded the clinical development of small molecule Stat3 inhibitors [3]. Present studies suggest H182 and H172 present favorable solubility in SIF and GIF environments, while metabolic stability may be modest. H182 shows favorable human liver microsome (HLM) and mouse liver microsome (MLM), half-life (t_1/2_) of 21 and 150 min, greater than the cut-off 15 min [41,42]. However, the human hepatocyte assay with CL_int_ 16 μL/min/10^6^ cells, indicates moderate to low stability (CL_int_ 19 μL/min/10^6^ cells). In addition, plasma stability was 10% and 23% after 2 h in human and mouse, respectively. Currently, these two parameters (hepatocyte and plasma stability) are being addressed to further improve the PK profile. H182 and H172 therefore represent suitable candidates for further PK optimization towards the goal of clinical development for treating TNBC and other tumors that are dependent on aberrantly-active Stat3 for the tumor phenotype. The azetidine-based small molecules are a new class of inhibitors that potently and irreversibly bind to Stat3, leading to tumor cell death and tumor growth inhibition *in vivo*. Industry standard CEREP SafetyScreen44 that showed a lack of inhibitory activities against a wide array of targets suggests a relatively low toxicity of the azetidine-based compounds.

### Statistical analysis.

Statistical analysis was performed on mean values using Prism GraphPad Software, Inc. (La Jolla, CA). The significance of differences between groups was determined by the paired *t-*test at *p < 0.05, or **p < 0.01.

## Supplementary Material

MMC2

MMC1

## Figures and Tables

**Table 1:** Replacement of *p*-fluorine with chlorine or hydrogen causes loss of STAT3 activity.

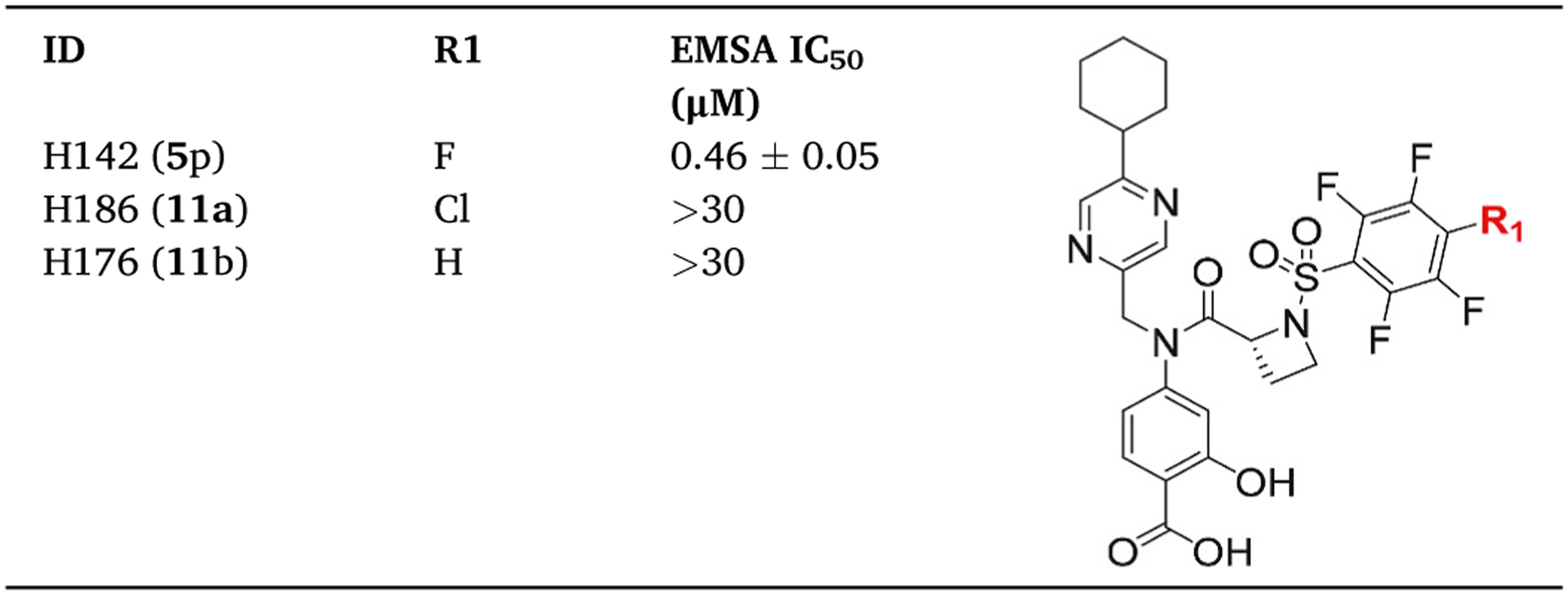

**Table 2:** Cysteine residues in Stat3 protein that were modified by azetidine compounds and detected by MS.

Compound name	Cys residue	*m/z*	Mr(expt)	Mr(calc)	ppm	Score	Expect	Peptide	Experimental reproducibility
H098	C328	920.3475	1838.6804	1838.6800	0.23	39	0.0002	F.VVERQPCMPM.H + 2 Oxidation (M); STAT3-H098 (C)	3 out of 3
	C426	791.2959	1580.5772	1580.5687	5.38	22	0.0093	N.GGRANCDASL.I + STAT3-H098 (C)	3 out of 3
H182	C468	1121.0018	2239.9891	2239.9880	0.51	37	0.00036	H.SLPVVVISNICQMPN.A + STAT3- H182 (C)	1 out of 2
	C542	711.2601	1420.5057	1420.4992	4.60	18	0.046	Y.SGCQITW.A + STAT3-H182 (C)	2 out of 2

**Table 3:** DMPK properties of H182 and H172.

	Solubility^a^			Metabolic Stability^a^			CYP Inhibition	Plasma Protein Binding	Plasma Stability	SafetyScreen44^b^
ID	PBS pH 7.4 (μg/mL)	SGF (μg/mL)	SIF (μg/mL)	MLM t_1/2_ (min)	HLM t_1/2_ (min)	Human Hepatocytes CL_int_ (μL/min/10^6^ cells)	IC_50_ or % Inh at 10 μM	(%)	(% Remaining)	Receptors over 80% Inhibition at 10 μM IC_50_ (μM)
H182	3.0	132	128	>150	21	14.6 (t_1/2_ 68 min)	CYP3A (Midazolam): IC_50_ 6.9 μM CYP3A (Testosterone): IC_50_ 1.2 μM CYP2C9: 33% CYP2D6: 9%	Human: 99.75 Mouse: 99.90	Human: 53% at 1 h 10% at 2 h Mouse: 69% at 1 h 23% at 2 h	hERG^c^ >10 GR 0.11 NET 0.40
H172	6.3	104	123	Nd	Nd	nd	nd	nd	nd	nd

nd, not determined.

a Cut-offs: Solubility >60 μg/mL; Metabolic Stability HLM half life >15 min, Human Hepatocytes CL_int_ 3.5–19.0 μL/min/10^6^ cells.

b SafetyScreen44^™^ according to Eurofins-CEREP Scientific.

c hERG IC_50_ results from Qpatch assay.

## Abbreviations

Stat: signal transducer and activator of transcription
PBST: phosphate-buffered saline tween-20
PBS: phosphate-buffered saline
EMSA: electrophoretic mobility shift assay
EGFR: epidermal growth factor receptor
JAK: Janus kinase
SH2: Src homology 2
MAPK: mitogen-activated protein kinase
Erk: extracellular signal-regulated kinase
FBS: fetal bovine serum
PARP: poly ADP-ribose polymerase
