# Supplementary material for: Novel potent azetidine-based compounds irreversibly inhibit Stat3 activation and induce antitumor response against human breast tumor growth in vivo
Source: Cancer Lett. Author manuscript; Available in PMC 2023 Jan 22. (PMC9867837; doi:10.1016/j.canlet.2022.215613)
Supplement: MMC2 [file NIHMS1791479-supplement-MMC2.pdf]

Supplementary Figures

Fig. S1

FP assay with rHis-STAT3(127-711) binding to 5-carboxy-fl-GpYLPQTV-NH_2 probe

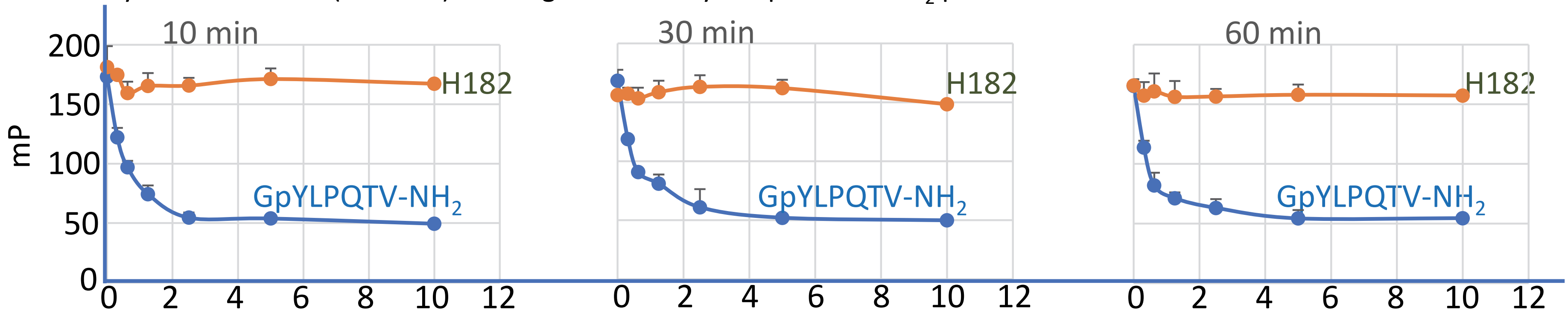

Supplementary Figures

Fig. S2

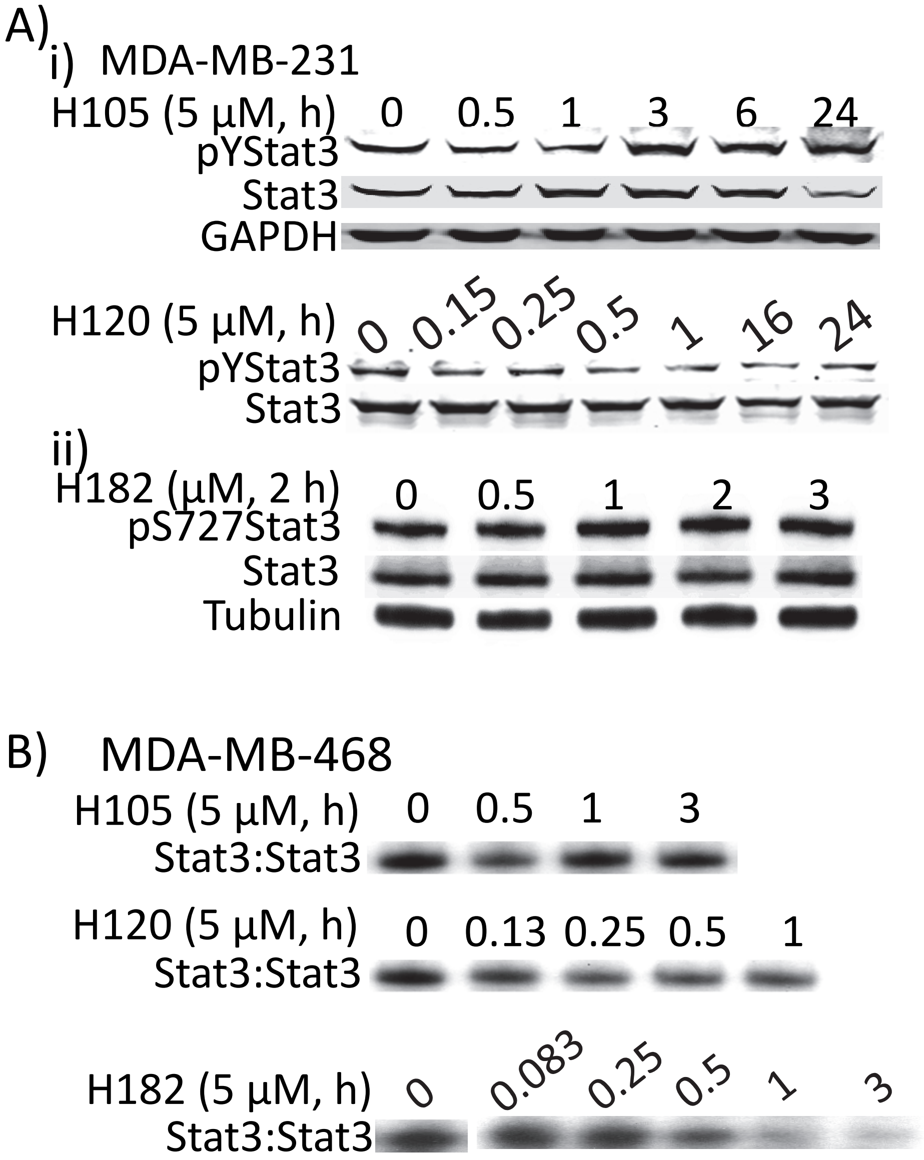

Fig. S3

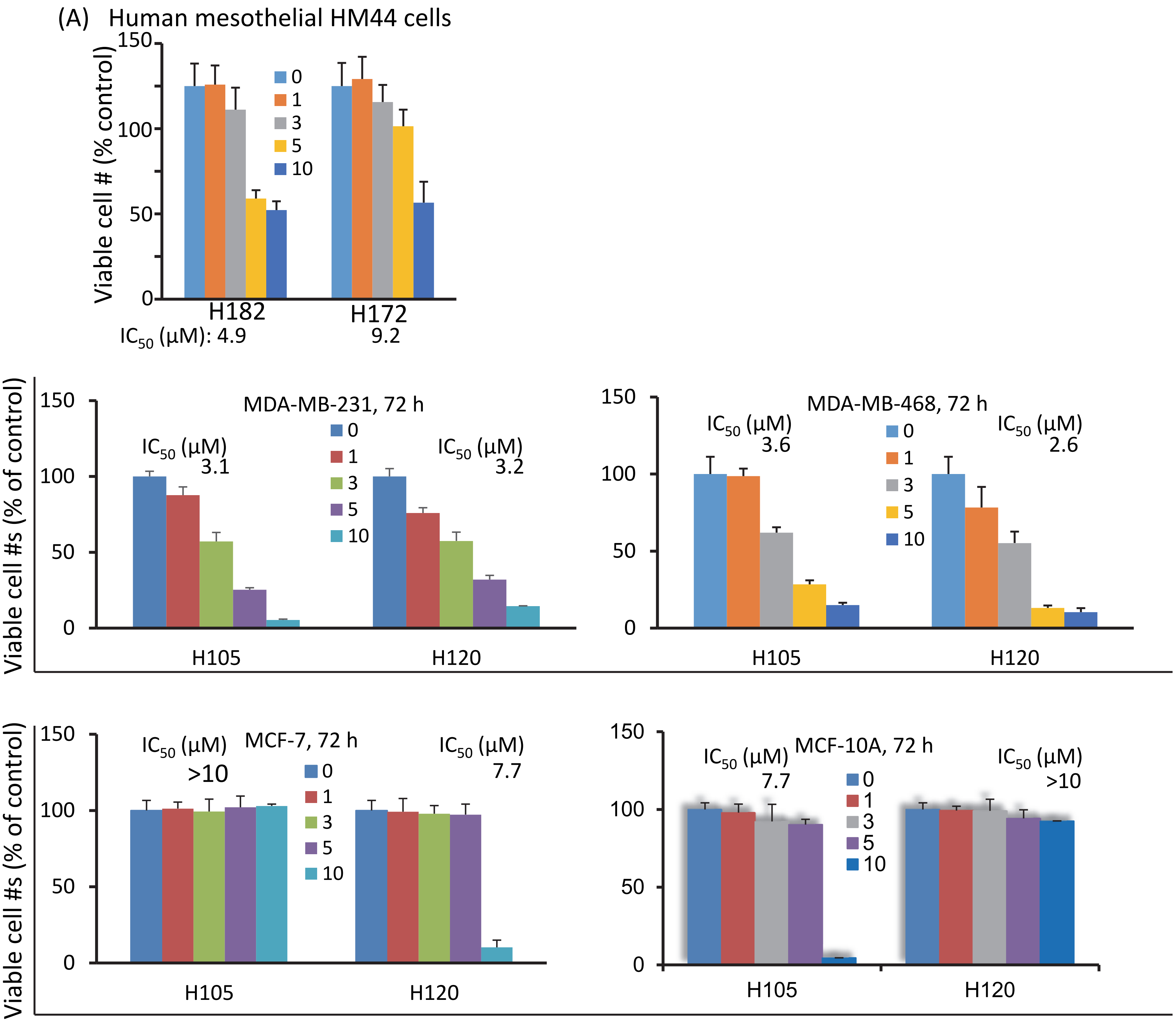
(A) Human mesothelial HM44 cells
Viable cell # (% control)
150
100
50
0
0
1
3
5
10
H182
H172
IC50 (μM): 4.9
9.2
B)
Viable cell #s (% of control)
MDA-MB-231, 72 h
IC50 (μM)
3.1
IC50 (μM)
3.2
H105
H120
MDA-MB-468, 72 h
IC50 (μM)
3.6
IC50 (μM)
2.6
C)
MCF-7, 72 h
IC50 (μM)
>10
IC50 (μM)
7.7
MCF-10A, 72 h
IC50 (μM)
7.7
IC50 (μM)
>10

Fig. S3

Supplementary Figures

D) Annexin V/PI – MDA-MB-231

Con | H182, 1 µM, 3 h | H182, 3 µM, 3 h

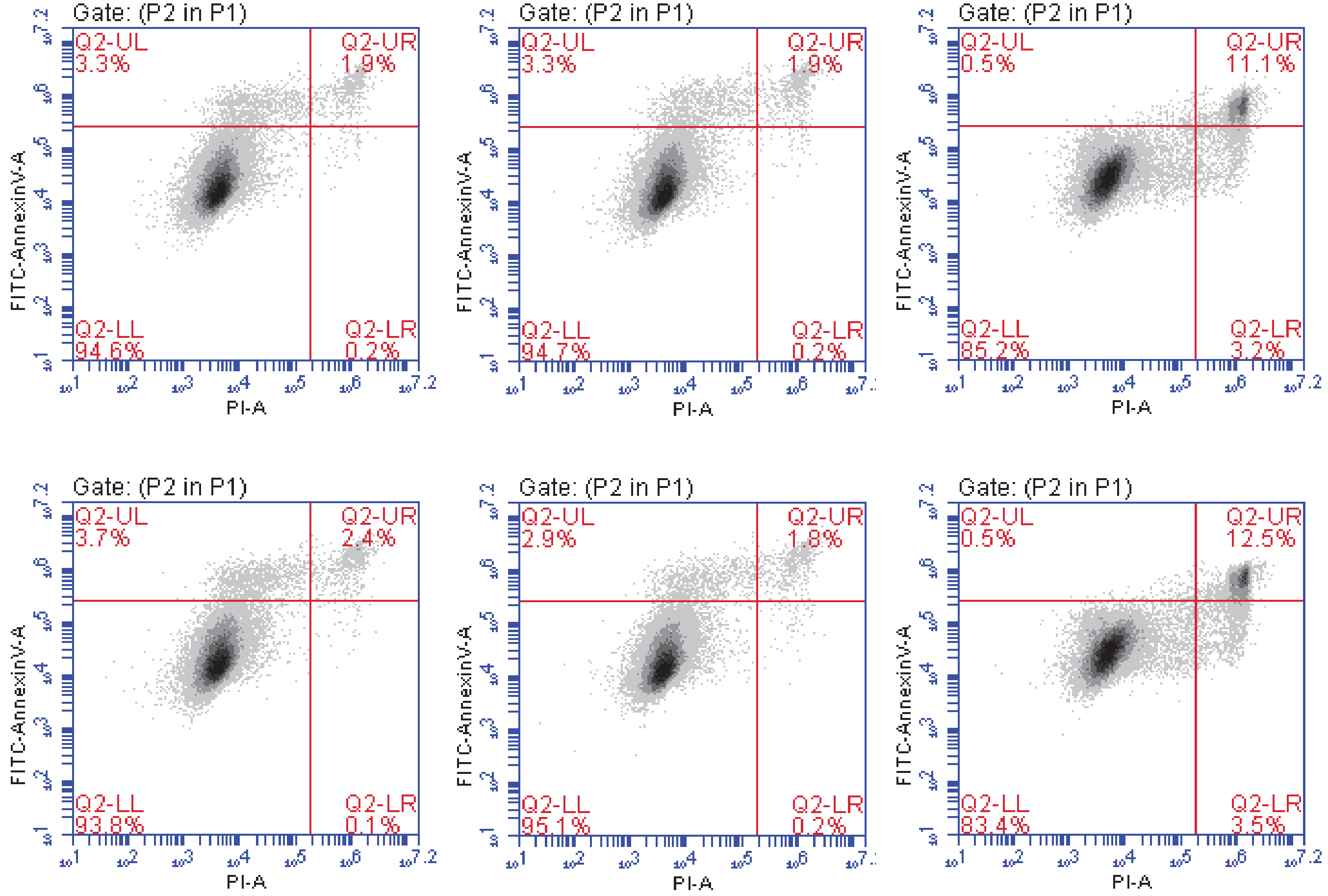

Supplementary Figures

Fig. S4 Regulation of downstream events

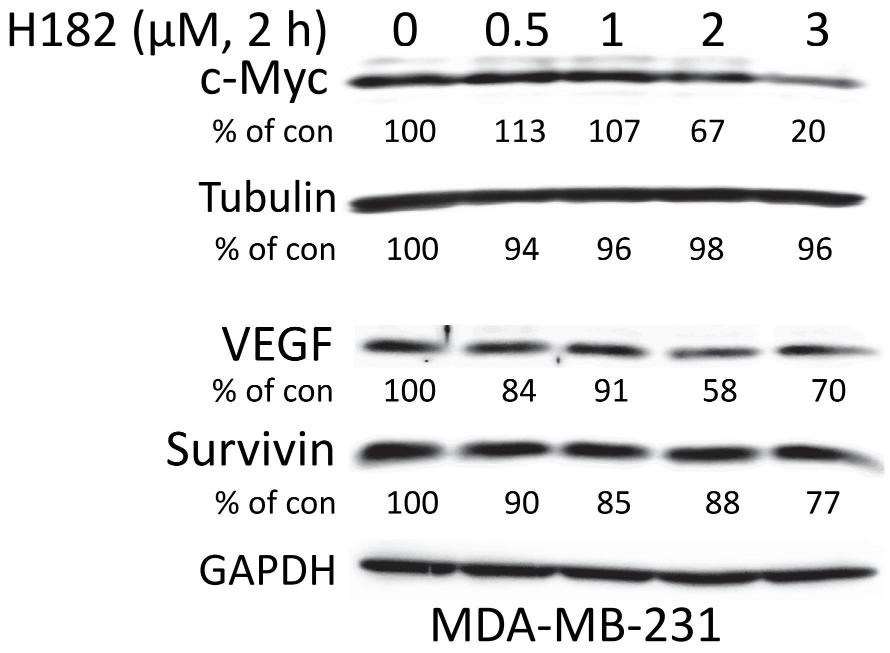

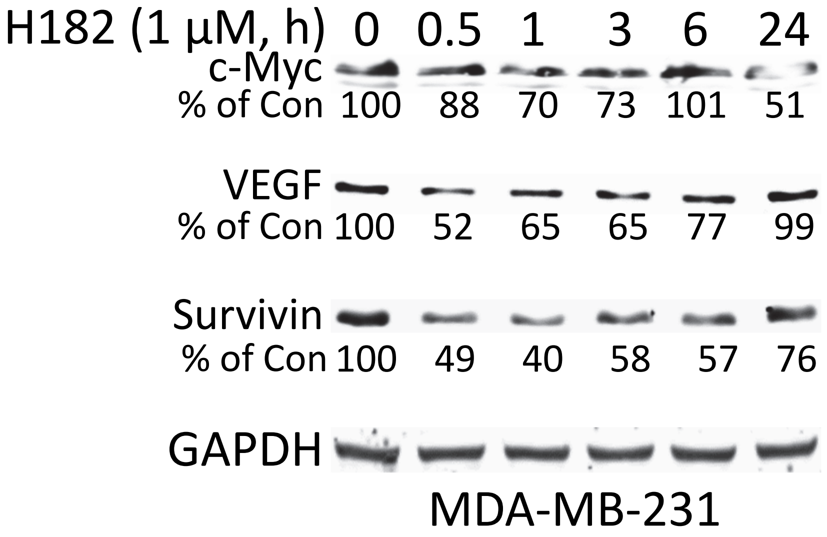

Fig. S5

Stat3 activity and target genes

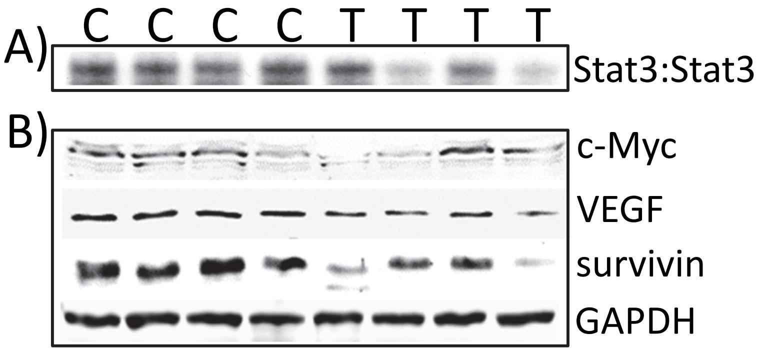

T, I.P. treatment; C, DMSO control
