# Supplementary material for: Novel potent azetidine-based compounds irreversibly inhibit Stat3 activation and induce antitumor response against human breast tumor growth in vivo
Source: Cancer Lett. Author manuscript; Available in PMC 2023 Jan 22. (PMC9867837; doi:10.1016/j.canlet.2022.215613)
Supplement: MMC1 [file NIHMS1791479-supplement-MMC1.pdf]

Supplementary Information

Novel potent azetidine-based compounds irreversibly inhibit Stat3 activation and induce antitumor response against human breast tumor growth *in vivo*

Peibin Yue[1,2], Yinsong Zhu[1,2], Christine Brotherton-Pleiss[3,4], Wenzhen Fu[3,4], Nagendra Verma[1,2], Jasmine Chen[3], Kayo Nakamura[4], Weiliang Chen[4], Yue Chen[1,2], Felix Alonso-Valenteen[2,5], Simoun Mikhael[2,5], Lali Medina-Kauwe[2,5], Kathleen M. Kershaw[2,6], Maria Celeridad[7], Songqin Pan[8], Allison S. Limpert[7], Douglas J. Sheffler[7], Nicholas D. P. Cosford[7], Stephen L. Shiao[2,6], Marcus A. Tius[3,4], Francisco Lopez-Tapia[1,2,*], and James Turkson[1,2,*]

[1]Department of Medicine, Division of Medical Oncology and [2]Cancer Biology Program, Cedars-Sinai Cancer, Cedars-Sinai Medical Center, 8700 Beverly Blvd, Los Angeles, CA, USA 90048, [3]Cancer Biology Program, University of Hawaii Cancer Center, 701 Ilalo St, Honolulu, HI, USA 96813, [4]Department of Chemistry, University of Hawaii, Manoa, 2545 McCarthy Mall, Honolulu, HI, USA 96825, [5]Department of Biomedical Sciences, Cedars-Sinai Medical Center, 8700 Beverly Blvd, Los Angeles, CA, USA 90048, [6]Department of Radiation Oncology, Cedars-Sinai Medical Center, 8700 Beverly Blvd, Los Angeles, CA, USA 90048, [7]Cell and Molecular Biology of Cancer Program, Cancer Center, Sanford Burnham Prebys Medical Discovery Institute, 10901 N. Torrey Pines Rd, La Jolla, CA, USA 92037, [8]W. M. Keck Proteomics Laboratory, University of California, Riverside, CA USA 92521

Running Title: Azetidine-based small molecule inhibitors of Stat3 activity

Keywords: Signal transducer and activator of transcription, small-molecule inhibitors, covalent modification, antitumor cell effects, tumor growth inhibition

Abbreviations: Stat, signal transducer and activator of transcription; PBST, phosphate-buffered saline tween-20; PBS, phosphate-buffered saline; EMSA, electrophoretic mobility shift assay; EGFR, epidermal growth factor receptor, JAK, Janus kinase, SH2, Src homology 2, MAPK, mitogen-activated protein kinase; Erk, extracellular signal-regulated kinase; FBS, fetal bovine serum; PARP, poly ADP-ribose polymerase.

*Corresponding author: James Turkson, Professor, Department of Medicine, Division of Hematology-Oncology, Samuel Oschin Comprehensive Cancer Institute, Cedars Sinai Medical Center, 8700 Beverly Blvd, Davis 5065, Los Angeles, CA, 90048, Tel. 310-423-6887; Email: james.turkson@cshs.org

1. Materials and Methods

1.1. Chemistry

1.1.1. Synthesis of H182 and H172

Final compounds are >95% pure by HPLC analysis.

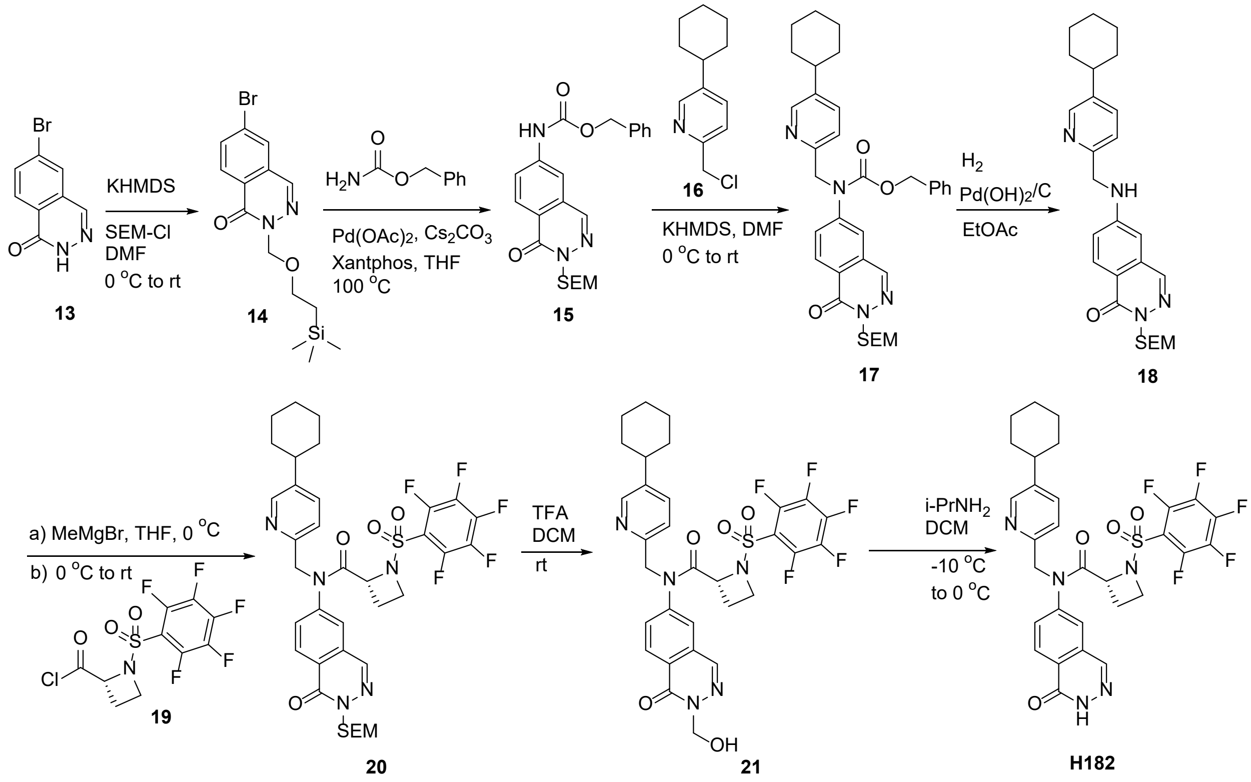

Scheme 1. Synthetic Preparation of H182

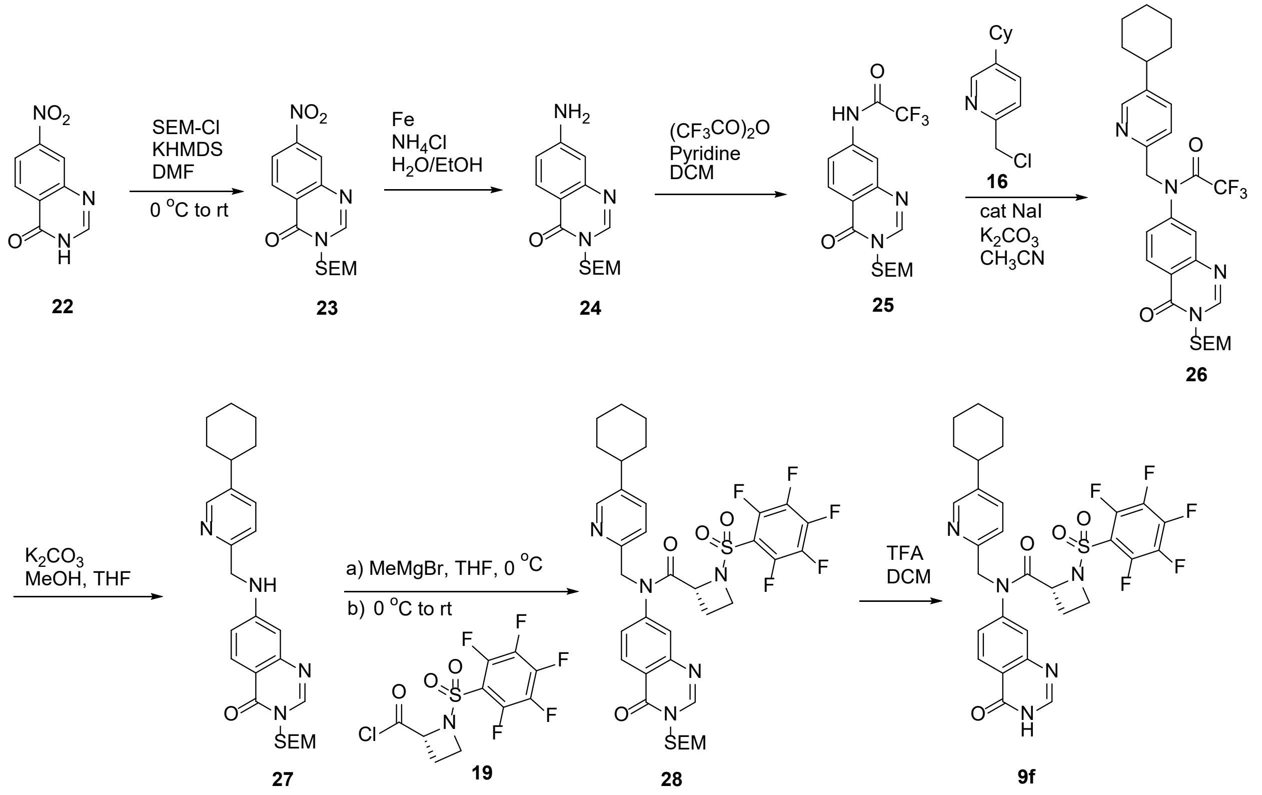

Scheme 2. Synthetic Preparation of H172 (9f)

Preparation of 6-bromo-2-((2-(trimethylsilyl)ethoxy)methyl)phthalazin-1(2H)-one **14**. To a solution of 6-bromophthalazin-1(2H)-one **13** (2.03 g, 9.02 mmol) in DMF (40 mL) was added at 0 ºC KHMDS (1M in THF, 10.8 mL, 10.8 mmol) under argon. After 10 minutes at 0 ºC, SEM-Cl (1.92 mL, 10.8 mmol) was added under argon. The mixture was allowed to reach rt and stirred for 26 h. Cold saturated ammonium chloride was added. The mixture was extracted with ethyl acetate (3 x). The extract was washed with water (2 x), brine, dried (Na_2SO_4) and concentrated to dryness to obtain 6-bromo-2-((2-(trimethylsilyl)ethoxy)methyl)phthalazin-1(2H)-one **14** (3.15 g), which was used as crude for next step. ^{1}H NMR (300 MHz, Chloroform-*d*) δ 8.33 (ddd, *J* = 8.4, 1.3, 0.7 Hz, 1H), 8.12 (d, *J* = 0.7 Hz, 1H), 7.93 – 7.85 (m, 2H), 5.57 (s, 2H), 3.84 – 3.62 (m, 1H), 1.10 – 0.89 (m, 1H), 0.01 (s, 9H).

Preparation of benzyl (1-oxo-2-((2-(trimethylsilyl)ethoxy)methyl)-1,2-dihydrophthalazin-6-yl)carbamate **15**. A mixture of crude 6-bromo-2-((2-(trimethylsilyl)ethoxy)methyl)phthalazin-1(2H)-one **14** (2.93 g, 8.25 mmol), (9,9-Dimethyl-9H-xanthene-4,5-diyl)bis(diphenylphosphane) (Xantphos) (0.477 g, 0.825 mmol), benzyl carbamate (1.87 g, 12.39 mmol), palladium acetate (0.185 g, 0.825 mmol) and cesium carbonate (5.37 g, 16.48 mmol) was thoroughly flushed with argon. Dioxane (103 mL) was added under argon. The mixture was heated at 100 °C (oil bath temperature) for 18 h. After cooling, the mixture was poured onto saturated ammonium chloride and filtered. The black solid was washed with ethyl acetate. The filtrate was extracted with ethyl acetate (2 x). The extract was washed with brine, dried (Na_2SO_4) and concentrated. Purification by flash column chromatography (8:2 to 7:3 hexane/ethyl acetate) gave benzyl (1-oxo-2-((2-(trimethylsilyl)ethoxy)methyl)-1,2-dihydrophthalazin-6-yl)carbamate **15** (1.62 g, 46% for 2 steps) as an off-white solid. ^{1}H NMR (300 MHz, Chloroform-*d*) δ 8.38 (d, *J* = 8.6 Hz, 1H), 8.15 (d, *J* = 0.7 Hz, 1H), 8.06 (d, *J* = 2.2 Hz, 1H), 7.50 (dd, *J* = 8.6, 2.2 Hz, 1H), 7.48 – 7.27 (m, 5H), 7.09 (bs, 1H), 5.57 (s, 2H), 5.27 (s, 2H), 3.81 – 3.68 (m, 2H), 1.06 – 0.94 (m, 2H), 0.0 (s, 9H).

Preparation of benzyl ((5-cyclohexylpyridin-2-yl)methyl)(1-oxo-2-((2-(trimethylsilyl)ethoxy)methyl)-1,2-dihydrophthalazin-6-yl)carbamate **17**. To a solution of benzyl (1-oxo-2-((2-(trimethylsilyl)ethoxy)methyl)-1,2-dihydrophthalazin-6-yl)carbamate **15** (894 mg, 2.1 mmol) in DMF (11.8 mL) was added at 0 °C KHMDS (1.0 M in THF, 2.72 mL, 2.72 mmol) under argon. After 5-10 minutes, 2-(chloromethyl)-5-cyclohexylpyridine **16** (1.0 M in toluene, 2.72 mL, 2.72 mmol) was added at 0 °C. The mixture was allowed to reach rt and stirred for 20 h. Cold saturated ammonium chloride was added, and the mixture was extracted with ethyl acetate (2 x). The extract was washed with water, brine, dried (Na_2SO_4) and concentrated. Purification by flash column chromatography (7:3 to 6:4 hexane/ethyl acetate) gave benzyl ((5-cyclohexylpyridin-2-yl)methyl)(1-oxo-2-((2-(trimethylsilyl)ethoxy)methyl)-1,2-dihydrophthalazin-6-yl)carbamate **17** (859 mg, 68% yield). ^{1}H NMR (300 MHz, Chloroform-*d*) δ 8.43 (d, *J* = 2.3 Hz, 1H), 8.37

(dd, J = 8.6, 0.8 Hz, 1H), 8.08 (s, 1H), 7.82 – 7.68 (m, 1H), 7.53 – 7.43 (m, 1H), 7.43 – 7.12 (m, 7H), 5.69 – 5.45 (m, 2H), 5.23 (d, J = 0.8 Hz, 2H), 5.10 (s, 2H), 3.88 – 3.56 (m, 2H), 2.54 (s, 1H), 1.84 (dd, J = 27.6, 10.8 Hz, 7H), 1.40 (q, J = 10.6 Hz, 4H), 1.08 – 0.68 (m, 2H), 0.0 (s, 9H).

Preparation of 6-(((5-cyclohexylpyridin-2-yl)methyl)amino)-2-((2-(trimethylsilyl)ethoxy)methyl)phthalazin-1(2H)-one **18**. To a solution of benzyl ((5-cyclohexylpyridin-2-yl)methyl)(1-oxo-2-((2-(trimethylsilyl)ethoxy)methyl)-1,2-dihydrophthalazin-6-yl)carbamate **17** (850 mg, 1.42 mmol) in ethyl acetate (8.2 mL) and methanol (8.2 mL) was added 10% $Pd(OH)_2/C$ (82.3 mg). A balloon filled with hydrogen was set up, and the mixture was stirred for 16 h. The mixture was filtered through Celite, and the filtrate was evaporated. Purification by flash chromatography column (6:4 to 45:55 hexane/ethyl acetate) gave 6-(((5-cyclohexylpyridin-2-yl)methyl)amino)-2-((2-(trimethylsilyl)ethoxy)methyl)phthalazin-1(2H)-one **18** as a white solid (515 mg, 78% yield). ^{1}H NMR (300 MHz, Chloroform-*d*) δ 8.47 (d, J = 2.2 Hz, 1H), 8.23 (d, J = 8.7 Hz, 1H), 8.01 (d, J = 0.8 Hz, 1H), 7.60 – 7.50 (m, 1H), 7.32 – 7.22 (m, 1H), 7.09 (ddd, J = 8.8, 2.4, 0.9 Hz, 1H), 6.67 (d, J = 2.3 Hz, 1H), 5.75 (bs, 1H), 5.54 (s, 2H), 4.53 (d, J = 4.9 Hz, 2H), 3.79 – 3.67 (m, 2H), 2.64-2.49 (m, 1H), 2.02-1.73 (m, 5H), 1.58-1.22 (m, 5H), 1.06 – 0.86 (m, 2H), 0.00 (s, 9H).

Preparation of (*R*)-*N*-((5-cyclohexylpyridin-2-yl)methyl)-*N*-(1-oxo-2-((2-(trimethylsilyl)ethoxy)methyl)-1,2-dihydrophthalazin-6-yl)-1-((perfluorophenyl)sulfonyl)azetidine-2-carboxamide **20**. To a solution of 6-(((5-cyclohexylpyridin-2-yl)methyl)amino)-2-((2-(trimethylsilyl)ethoxy)methyl)phthalazin-1(2H)-one **18** (497 mg, 1.07 mmol) in THF (8.5 mL) was added at 0 ºC methylmagnesium bromide (1.4 M in THF, 1.92 mL, 2.67 mmol) under argon. After 10 minutes at 0 ºC, powder (*R*)-1-((perfluorophenyl)sulfonyl)azetidine-2-carbonyl chloride **19** (560 mg, 1.61 mmol) was added at 0 ºC. The mixture was allowed to reach room temperature and stirred for one hour. Cold aqueous saturated ammonium chloride was added followed by

water. The mixture was extracted with ethyl acetate (2 x). The extract was washed with brine, dried (Na_2SO_4) and concentrated. Purification by flash column chromatography (1:1 hexane/ethyl acetate) gave (*R*)-*N*-((5-cyclohexylpyridin-2-yl)methyl)-*N*-(1-oxo-2-((2-(trimethylsilyl)ethoxy)methyl)-1,2-dihydrophthalazin-6-yl)-1-((perfluorophenyl)sulfonyl)azetidine-2-carboxamide **20** (720 mg, 87% yield). ^{1}H NMR (300 MHz, Chloroform-*d*) δ 8.46 (d, *J* = 8.4 Hz, 1H), 8.35 (s, 1H), 8.13 (s, 1H), 7.66 – 7.47 (m, 3H), 7.25 – 7.11 (m, 1H), 5.58 (s, 2H), 5.09 – 4.85 (m, 3H), 4.23 – 4.01 (m, 2H), 3.81 – 3.69 (m, 2H), 2.53 (s, 1H), 2.44 – 2.24 (m, 1H), 2.12 – 1.95 (m, 1H), 1.94 – 1.70 (m, 5H), 1.52 – 1.12 (m, 5H), 1.07 – 0.95 (m, 2H), 0.01 (s, 9H). HRMS (ESI+) m/z 778.2513 (calculated), 778.2611 (found) $[M + H]^+$.

Preparation of (*R*)-*N*-((5-cyclohexylpyridin-2-yl)methyl)-*N*-(2-(hydroxymethyl)-1-oxo-1,2-dihydrophthalazin-6-yl)-1-((perfluorophenyl)sulfonyl)azetidine-2-carboxamide **21**. To a solution of (*R*)-*N*-((5-cyclohexylpyridin-2-yl)methyl)-*N*-(1-oxo-2-((2-(trimethylsilyl)ethoxy)methyl)-1,2-dihydrophthalazin-6-yl)-1-((perfluorophenyl)sulfonyl)azetidine-2-carboxamide **20** (714 mg (0.918 mmol) in dichloromethane (14 mL) was added trifluoroacetic acid (4.5 mL) under argon. The mixture was stirred for two hours. The mixture was poured onto cold aqueous 10% sodium bicarbonate and extracted with dichloromethane (2 x). The aqueous layer pH was 7-8. The organic phase was washed with additional aqueous 10% sodium bicarbonate, dried (Na_2SO_4) and concentrated. Purification by flash column chromatography (7:3 to 1:1 hexane/acetone) gave as light yellow foam (*R*)-*N*-((5-cyclohexylpyridin-2-yl)methyl)-*N*-(2-(hydroxymethyl)-1-oxo-1,2-dihydrophthalazin-6-yl)-1-((perfluorophenyl)sulfonyl)azetidine-2-carboxamide **21** (569 mg, 91% yield). ^{1}H NMR (300 MHz, Chloroform-*d*) δ 8.49 – 8.32 (m, 2H), 8.19 – 8.04 (m, 1H), 7.72 – 7.46 (m, 3H), 7.31 – 7.17 (m, 1H), 5.66 (s, 2H), 5.12 – 4.84 (m, 3H), 4.19 – 4.07 (m, 2H), 2.62 – 2.45 (m, 1H), 2.43 – 2.26 (m, 1H), 2.12 – 1.95 (m, 1H), 1.94 – 1.73 (m, 5H), 1.48 – 1.35 (m, 5H). MS (ESI+) m/z 678.3 $[M + H]^+$.

Preparation of (*R*)-*N*-((5-cyclohexylpyridin-2-yl)methyl)-*N*-(1-oxo-1,2-dihydrophthalazin-6-yl)-1-((perfluorophenyl)sulfonyl)azetidine-2-carboxamide **H182**. To a solution of (*R*)-*N*-((5-cyclohexylpyridin-2-yl)methyl)-*N*-(2-(hydroxymethyl)-1-oxo-1,2-dihydrophthalazin-6-yl)-1-((perfluorophenyl)sulfonyl)azetidine-2-carboxamide **21** (533 mg, 0.787 mmol) in dichloromethane (8.8 mL) was added at -10 ºC isopropylamine (0.14 mL, 1.58 mmol) under argon. The mixture was stirred at 0 ºC for 24 hours. Cold aqueous acetic acid/sodium acetate buffer (12 mL) was added. The mixture was extracted with dichloromethane (2 x). The extract was washed with water, dried (Na_2SO_4) and concentrated to dryness. Purification by flash column chromatography (6:4 hexane/acetone), followed by repurification of the purest fraction by preparative thin layer chromatography (6:4 hexane/acetone) gave (*R*)-*N*-((5-cyclohexylpyridin-2-yl)methyl)-*N*-(1-oxo-1,2-dihydrophthalazin-6-yl)-1-((perfluorophenyl)sulfonyl)azetidine-2-carboxamide **H182** (225 mg, 100% pure by HPLC) as a white foam. ^{1}H NMR (300 MHz, Chloroform-*d*) δ 11.55 (s, 1H), 8.42 (s, 1H), 8.33 (d, *J* = 8.4 Hz, 1H), 8.14 – 7.98 (m, 1H), 7.67 – 7.43 (m, 3H), 7.40 – 7.24 (m, 1H), 5.19 – 4.80 (m, 3H), 4.19 – 4.02 (m, 2H), 2.63 – 2.45 (m, 1H), 2.43 – 2.24 (m, 1H), 2.11 – 1.60 (m, 7H), 1.56 – 1.15 (m, 5H). HRMS (ESI+) m/z 648.1699 (calculated), 648.1850 (found) $[M + H]^+$.

Preparation of 7-nitro-3-((2-(trimethylsilyl)ethoxy)methyl)quinazolin-4(3H)-one **23**. To a suspension of 7-nitroquinazolin-4(3H)-one **22** (196 mg, 1.03 mmol) in DMF (8 mL) was added at 0 ºC KHMDS (1M in THF, 1.23 mL, 1.23 mmol) under nitrogen. After 5-10 min at 0 ºC, SEM-Cl (0.22 mL, 1.23 mmol) was added dropwise. The resulting homogeneous mixture was allowed to reach rt and stirred for 3 hours. Cold saturated ammonium chloride was added followed by water. The mixture was extracted with ethyl acetate (2 x). The extract was washed with water, brine, dried (sodium sulfate) and concentrated. Purification by column chromatography (8:2 hexane/ethyl acetate) gave 7-nitro-3-((2-(trimethylsilyl)ethoxy)methyl)quinazolin-4(3H)-one **23** as yellow solid (227 mg, 70% yield). ^{1}H NMR (300

MHz, CDCl3) δ 8.59 (m, 1H), 8.51 (d, J = 8.8 Hz, 1H), 8.37 – 8.22 (m, 2H), 5.48 (s, 2H), 3.83 – 3.62 (m, 2H), 1.08 – 0.92 (m, 2H), 0.07 – -0.03 (m, 9H).

Preparation of 7-amino-3-((2-(trimethylsilyl)ethoxy)methyl)quinazolin-4(3H)-one **24**. To 7-nitro-3-((2-(trimethylsilyl)ethoxy)methyl)quinazolin-4(3H)-one **23** (103.8 mg, 0.323 mmol) and ammonium chloride (176 mg, 3.29 mmol)were added ethanol (2.4 mL) and water (1.2 mL) under argon. Iron powder (126 mg, 2.25 at Eq), and the mixture was stirred vigorously and heated at 66 °C overnight. After cooling, the mixture was filtered through Celite®. The cake was washed with ethyl acetate. Water was added to the filtrate, and the mixture was extracted with ethyl acetate (2 x). The extract was washed with brine, dried (Na_2SO_4) and concentrated to dryness to obtain 7-amino-3-((2-(trimethylsilyl)ethoxy)methyl)quinazolin-4(3H)-one **24** (102 mg) as a cream solid, which was taken as crude to next step. ^{1}H NMR (300 MHz, $CDCl_3$) δ 8.17 – 8.04 (m, 2H), 6.91 – 6.76 (m, 2H), 5.41 (s, 2H), 4.53 – 3.92 (m, 2H), 3.78 – 3.60 (m, 2H), 1.06 – 0.89 (m, 2H), -0.01 (s, 9H).

Preparation of 2,2,2-trifluoro-*N*-(4-oxo-3-((2-(trimethylsilyl)ethoxy)methyl)-3,4-dihydroquinazolin-7-yl)acetamide **25**. To a solution of 7-amino-3-((2-(trimethylsilyl)ethoxy)methyl)quinazolin-4(3H)-one **24** (146 mg, 0.501 mmol) in dichloromethane (2.7 mL) was added at 0 °C pyridine (0.09 mL, 1.10 mmol), followed by trifluoroacetic anhydride (0.078 mL, 0.551 mmol) under argon. The mixture was allowed to reach room temperature and stirred for 2 hours. The mixture was diluted with dichloromethane and washed with pH 2 buffer solution, aqueous saturated sodium bicarbonate, dried (Na_2SO_4) and concentrated to dryness. Purification by flash column chromatography (65:35 hexane/ethyl acetate) gave as white solid 2,2,2-trifluoro-*N*-(4-oxo-3-((2-(trimethylsilyl)ethoxy)methyl)-3,4-dihydroquinazolin-7-yl)acetamide **25** (184 mg, 95% yield for two steps). ^{1}H NMR (300 MHz, $CDCl_3$) δ 8.37 (d, J = 8.7 Hz, 1H), 8.18 (m, 2H),

8.02 (d, J = 2.1 Hz, 1H), 7.75 (dd, J = 8.7, 2.1 Hz, 1H), 5.45 (s, 2H), 3.81 – 3.60 (m, 2H), 1.07 – 0.91 (m, 2H), 0.09 – -0.03 (m, 9H).

Preparation of N-((5-cyclohexylpyridin-2-yl)methyl)-2,2,2-trifluoro-*N*-(4-oxo-3-((2-(trimethylsilyl)ethoxy)methyl)-3,4-dihydroquinazolin-7-yl)acetamide **26**. A mixture of 2,2,2-trifluoro-*N*-(4-oxo-3-((2-(trimethylsilyl)ethoxy)methyl)-3,4-dihydroquinazolin-7-yl)acetamide **25** (225 mg, 0.58 mmol), potassium carbonate (161 mg, 1.16 mmol) and sodium iodide (17.5 mg, 0.116 mmol) was thoroughly flushed with argon. Acetonitrile (7.2 mL) and 2-(chloromethyl)-5-cyclohexylpyridine **16** (0.5 M in toluene, 1.2 mL) were added under argon. The mixture was heated at 65 ^{o}C for 7.5 h, when additional 2-(chloromethyl)-5-cyclohexylpyridine (0.5 M in toluene, 1.2 mL) was added. The reaction was maintained at 65 ^{o}C for a total of 24 h. After cooling, saturated ammonium chloride was added, followed by water. The mixture was extracted with ethyl acetate (2 x). The extract was washed with brine, dried (Na_2SO_4) and concentrated. Purification by flash column chromatography (8:2 hexane/acetone) furnished N-((5-cyclohexylpyridin-2-yl)methyl)-2,2,2-trifluoro-*N*-(4-oxo-3-((2-(trimethylsilyl)ethoxy)methyl)-3,4-dihydroquinazolin-7-yl)acetamide **26** (203 mg, 62% yield). ^{1}H NMR (300 MHz, $CDCl_3$) δ 8.40 (d, J = 2.3 Hz, 1H), 8.33 (d, J = 8.3 Hz, 1H), 8.18 (s, 1H), 7.65 (d, J = 2.3 Hz, 1H), 7.55 (d, J = 8.3 Hz, 1H), 7.47 – 7.36 (m, 1H), 7.36 – 7.27 (m, 1H), 5.44 (s, 2H), 5.10 (s, 2H), 3.80 – 3.60 (m, 2H), 2.67 – 2.45 (m, 1H), 2.1 – 1.6 (m, 5H), 1.55 – 1.15 (m, 5H), 1.07 – 0.88 (m, 2H), 0.02 (s, 9H).

Preparation of 7-(((5-cyclohexylpyridin-2-yl)methyl)amino)-3-((2-(trimethylsilyl)ethoxy)methyl)quinazolin-4(3H)-one **27**. To a solution of N-((5-cyclohexylpyridin-2-yl)methyl)-2,2,2-trifluoro-*N*-(4-oxo-3-((2-(trimethylsilyl)ethoxy)methyl)-3,4-dihydroquinazolin-7-yl)acetamide **26** (203 mg, 0.363 mmol) in THF (1.9 mL) and methanol (2.3 mL) was added potassium carbonate (100 mg, 0.73 mmol) under argon. The mixture was stirred at room temperature for 2 hours.

Aqueous saturated ammonium chloride was added followed by water. The mixture was extracted with ethyl acetate (2 x). The extract was washed with brine, dried (Na_2SO_4) and concentrated. Purification by flash column chromatography (1:1 to 25:75 hexane/ethyl acetate) gave 7-(((5-cyclohexylpyridin-2-yl)methyl)amino)-3-((2-(trimethylsilyl)ethoxy)methyl)quinazolin-4(3H)-one **27** (147 mg, 88% yield). ^{1}H NMR (300 MHz, CDCl3) δ 8.46 (d, *J* = 2.2 Hz, 1H), 8.10 (m, 2H), 7.58 (dd, *J* = 8.0, 2.2 Hz, 1H), 7.32 (d, *J* = 8.0 Hz, 1H), 6.88 (dd, *J* = 8.8, 2.3 Hz, 1H), 6.77 (d, *J* = 2.3 Hz, 1H), 5.89 -5.69 (br, 1H), 5.40 (s, 2H), 4.56 (s, 2H), 3.77 – 3.61 (m, 2H), 2.66 – 2.46 (m, 1H), 2.0 – 1.70 (m, 5H), 1.58 – 1.14 (m, 5H), 1.05 – 0.86 (m, 2H), 0.07 (s, 9H).

Preparation of (*R*)-*N*-((5-cyclohexylpyridin-2-yl)methyl)-*N*-(4-oxo-3-((2-(trimethylsilyl)ethoxy)methyl)-3,4-dihydroquinazolin-7-yl)-1-((perfluorophenyl)sulfonyl)azetidine-2-carboxamide **28**. To a solution of 7-(((5-cyclohexylpyridin-2-yl)methyl)amino)-3-((2-(trimethylsilyl)ethoxy)methyl)quinazolin-4(3H)-one **27** (129 mg, 0.28 mmol) in THF (2.2 mL) was added at 0 ºC MeMgBr (1.4 M in THF, 0.50 mL) under argon. After 5-10 minutes, powder (*R*)-1-((perfluorophenyl)sulfonyl)azetidine-2-carbonyl chloride **19** (146 mg, 0.42 mmol) was added at 0 ºC. The mixture was allowed to reach rt and stirred for 1 hour. Cold ammonium chloride was added followed by water. The mixture was extracted with EtOAc (2 x). The extract was washed with brine, dried (sodium sulfate), and concentrated. Purification by column chromatography (1:1 to 3:7 hexane/ethyl acetate) gave (*R*)-*N*-((5-cyclohexylpyridin-2-yl)methyl)-*N*-(4-oxo-3-((2-(trimethylsilyl)ethoxy)methyl)-3,4-dihydroquinazolin-7-yl)-1-((perfluorophenyl)sulfonyl)azetidine-2-carboxamide **28** (185 mg, 86% yield). ^{1}H NMR (300 MHz, CDCl3) δ 8.43 – 8.26 (m, 2H), 8.18 (s, 1H), 7.55 (s, 1H), 7.42 – 7.10 (m, 3H), 5.45 (s, 2H), 5.21 – 4.90 (m, 3H), 4.27 – 3.97 (m, 2 H), 3.84 – 3.51 (m, 2H), 2.66 – 2.48 (m, 1H), 2.48 – 2.27 (m, 1H), 1.96 – 1.71 (m, 6H), 1.54 – 1.13 (m, 5H), 1.10 – 0.89 (m, 2H), 0.03 (s, 9H).

Preparation of (*R*)-*N*-((5-cyclohexylpyridin-2-yl)methyl)-*N*-(4-oxo-3,4-dihydroquinazolin-7-yl)-1-((perfluorophenyl)sulfonyl)azetidine-2-carboxamide H172 (**9f**). To a solution of (*R*)-*N*-((5-cyclohexylpyridin-2-yl)methyl)-*N*-(4-oxo-3-((2-(trimethylsilyl)ethoxy)methyl)-3,4-dihydroquinazolin-7-yl)-1-((perfluorophenyl)sulfonyl)azetidine-2-carboxamide **28** (183 mg, 0.24 mmol) in dichloromethane (1 mL) was added trifluoroacetic acid (1 mL) under argon. The mixture was stirred at rt for 2 h. Additional dichloromethane was added, and the mixture was poured onto cold saturated sodium bicarbonate. After separation of phases, the aqueous layer was extracted with additional dichloromethane. The combined organics was washed with additional saturated sodium bicarbonate, dried (sodium sulfate) and concentrated. Purification by column chromatography (100% ethyl acetate to 95:5 ethyl acetate/methanol) gave (*R*)-*N*-((5-cyclohexylpyridin-2-yl)methyl)-*N*-(4-oxo-3,4-dihydroquinazolin-7-yl)-1-((perfluorophenyl)sulfonyl)azetidine-2-carboxamide H172 (128 mg, 84% yield, >95% pure by HPLC). ^{1}H NMR (300 MHz, Chloroform-*d*) δ 11.51 (bs, 1H), 8.37 (d, *J* = 2.2 Hz, 1H), 8.23 (d, *J* = 8.4 Hz, 1H), 7.92 (s, 1H), 7.65 – 7.49 (m, 2H), 7.43 – 7.28 (m, 2H), 5.22 – 4.89 (m, 3H), 4.21 – 4.02 (m, 2H), 2.67 – 2.47 (m, 1H), 2.45 – 2.26 (m, 1H), 2.18 – 1.98 (m, 1H), 1.97 – 1.67 (m, 5H), 1.50 – 1.18 (m, 5H). HRMS (ESI+) m/z 648.1699 (calculated), 648.1847 (found) $[M + H]^{+}$.

1.2. Biology

1.2.1. Nuclear extract preparation, gel shift assays, and densitometric analysis

These studies were carried out as previously described [1-3] using the ^{32}P-labeled oligonucleotide probes, hSIE (high affinity sis-inducible element from the *c-fos* gene, m67 variant, 5'-AGCTTCATTTCCCGTAAATCCCTA) that binds Stat1 and Stat3 [1, 2] and the mammary gland factor element (MGFe) from the bovine β–casein gene promoter (sense strand, 5'-AGATTTCTAGFAATTCAA) that binds Stat5 and Stat1 [1, 2]. Briefly, nuclear extracts containing activated Stats prepared from NIH3T3/v-Src or the EGF-stimulated NIH3T3/hEGFR fibroblasts were pre-incubated with increasing

concentration of H120, H105, H172 or H182 at room temperature for 30 min, or in some cases for 10 or 60 min, prior to incubation with the radiolabeled probe for 30 min at 30 °C and subjecting to EMSA analysis. In another case, the nuclear extract was pre-incubated with the radiolabeled probe for 30 min at 30 °C prior to incubation with increasing concentration of H182 at room temperature for 30 min and subjecting to EMSA analysis. Bands corresponding to Stats DNA-binding activities were scanned and quantified using ImageJ and plotted as a percentage of control (vehicle) against the concentration of the inhibitor, from which the IC_{50} values were derived [1, 2].

1.2.2. Luciferase reporter assay

These studies were performed as previously reported [1, 2, 4, 5] using the v-Src-transformed NIH3T3/v-Src fibroblasts [1-5]. Cells in culture in 12-well plates were transiently transfected. The next day, the transfected cells were treated with 0-3 μM of H182 for 1 or 3 h, after which cells were harvested, and cytosolic extracts were prepared for luciferase reporter assay, which was performed according to the manufacturer's instructions (Promega, Madison, WI). Luciferase activity was normalized to total protein concentration of cell lysate, which was determined by Bradford assay (Bio-Rad, Hercules, CA).

1.2.3. Site directed mutagenesis to create STAT3 mutants

The prokaryotic pET28-hStat3(127-711)-6×His plasmid construct that contains the DNA fragment coding human Stat3 protein residues 127-711 (referring to NM_139276.3) with a C-terminal 6×His tag, which is flanked by restriction sites NdeI and XhoI, was kindly provided by Dr. Yuan Chen (University of California, San Diego) [6]. The construct was used to generate Cysteine (Cys or C) to Alanine (A) or Serine (S) mutants of the wild-type recombinant Stat3(127-711)-6×His (wtStat3) protein at Cys residues C328A (DNA codon: *TG*C to *GC*C, same below), C426A (*TG*T to *GC*T), C468A (*TG*T to *GC*T), and C542S (T*G*T to T*C*T) by

using QuikChange II XL Site-Directed Mutagenesis Kit (Agilent Technologies, Santa Clara, CA) following the manufacturer's Instructions, and the mutated constructs were verified by DNA Sequencing.

1.2.4. Protein expression and purification

The un-phosphorylated His-tagged recombinant wtStat3 protein was expressed in BL21(DE3) bacterial cells induced by 0.01 mM IPTG at 18 °C overnight. To express tyrosine (Tyr, Y)-phosphorylated recombinant Stat3 (pYStat3, both wt and Cys mutants), each plasmid was transformed into chemically competent tyrosine kinase derivative of the Escherichia coli BL21(DE3) TKB1 strain (Agilent Technologies), which harbors an inducible tyrosine kinase that can phosphorylate the Tyr residues of Stat3 protein. A two-step induction protocol was followed during the expression procedure according to the instructions. Bacterial cells were grown at 37°C to a density of A_{600} = 0.6 – 1.0, following which IPTG was added for the induction to a final concentration of 0.4 mM for 3 h. Then cells expressing Stat3 protein were harvested and resuspended in a TK induction medium containing 12.5 μg/ml indole acrylic acid. The tyrosine kinase-expressing culture was then harvested after 2 h of incubation at 37°C, resuspended in lysis buffer (20 mM HEPES, 100 mM KCl, 10% glycerol, 1 mM EDTA and 20 mM DTT), and stored at -80°C. The bacterial cell resuspension in lysis buffer was sonicated on ice, and the Stat3 protein was purified by using ammonium sulfate precipitation and nickel-affinity chromatography with GE HisTrap FF crude columns (Cytiva, Marlborough, MA). The eluted proteins were dialyzed overnight at 4°C in a buffer containing 20 mM HEPES, 250 mM KCl and 2 mM DTT, and the dialyzed proteins were concentrated using centrifugal filter devices (Amicon-15, 10,000 molecular weight cut-offs; Millipore Sigma; Burlington, MA). The freshly prepared protein was aliquoted, flash-frozen in liquid nitrogen, and stored at -80°C for later use.

1.2.5. SDS-PAGE/Western blotting analysis

These studies were performed as previously described [1-3]. Briefly, snap-frozen tumor tissues or cultured cells treated or not were harvested and whole-cell or tissue lysates were prepared in radioimmunoprecipitation assay (RIPA) buffer. Samples of equal total protein were subjected to SDS-PAGE and immunoblotting analysis. Primary antibodies used were anti- Stat3, pY705-Stat3, pS727Stat3, p-Stat1, Stat1, pY1173EGFR, EGFR, pY1007/1008JAK2, JAK2, pY416Src, Src, Shc, p-Shc, pS473Akt, Akt, pT202/Y204Erk1/2 (p44/42), Erk1/2, PARP, caspase 3, c-Myc, survivin, VEGF, β-actin, α/β-tubulin, and GAPDH. For the studies of the effects of cell density and confluence on pY705Stat3, cells were seeded at a density of 1.4×10^{4} cells/cm^{2} in culture dishes. The next day, cells were untreated (DMSO control, 0.1%) or treated at the same time with 1 or 2 μM H182, allowed to incubate for the indicated time and then harvested for whole-cell lysate preparation and SDS-PAGE / immunoblotting analysis. After 48-h incubation, the cell confluency of the DMSO-treated samples reached to around 60% for MDA-MB-468 cells and 80% for MDA-MB-231 cells.

1.2.6. Cell viability assays

CyQuant assay was performed to evaluate compounds as previously reported [1-3]. Briefly, cells (5×10^{3} per well, 100 μl) in culture in 96-well plates were treated once with 0-10 μM concentrations of H120, H105, H172 or H182 alone, or in the case of combination treatment, first pre-treated with a single, 1 μM concentration of H182 for 6 h and then treated with 0-100 nM docetaxel or 0-20 μM cisplatin for a total of 72 h. Viable cell numbers were assessed by a CyQuant cell proliferation kit according to the manufacturer's instructions (Thermo/Life Technologies Corp, Carlsbad, CA). Viable cell numbers were normalized to that of DMSO (control)-treated samples.

1.2.7. Soft-agar colony formation and clonogenic survival assays

These studies were performed as previously reported [1-3]. For the clonogenic survival assay, briefly, cells were seeded as single-cell (500 cells per well) in 6-well plates, then untreated (DMSO control) or treated once the following day with H182 and allowed to grow until large colonies were visible, which were stained with crystal violet for 4 h and imaged with a FluorChem imaging system (Protein Simple, Santa Clara, CA). For the soft-agar assay, briefly, each well contained a bottom layer of 1 ml of 1% agarose in complete DMEM and a top layer of 1 ml of 0.5% agarose in complete DMEM containing 500 cells. After the upper agar layer containing cells solidified, 1 ml culture medium containing H182 (0-2 μM) or DMSO (control) was overlaid, and the cultures were maintained at 37 ^{o}C in the incubator, with the medium replacement every 3-4 days with fresh medium that contains the inhibitor or not. Cells were allowed to culture for 3-4 weeks until large colonies were visible under microscope. The colonies were stained with crystal violet and counted.

1.2.8. Confocal microscopy studies

These studies were performed as previously reported [5]. Single cells (2×10^{4} cells in 1 ml) in logarithmic growth phase were seeded in 35-mm μ-Dishes (iBidi, Germany). On the next day, cells were treated with H182 or DMSO (control) for 12 h. Cells were washed twice with ice-cold PBS, fixed with 4% paraformaldehyde for 20 min at room temperature (RT), and permeabilized with 0.3% Triton X-100 in PBS at RT for 20 min. The samples were blocked with 5% bovine serum albumin (BSA) in phosphate-buffered saline (PBS) for 1 h at RT and incubated with rabbit anti-Stat3 monoclonal antibody (1:100 dilution, Cell Signaling, # 4904S) in PBS with 1% BSA overnight at 4°C. Then the samples were washed three times with PBS and incubated with Alexa Fluor 594-conjugated anti-rabbit secondary antibody (1:100 dilution, Thermo-Fisher) in PBS with 1% BSA in the dark for 1 h at RT, washed again with PBS and mounted with mounting medium containing DAPI (Vector Laboratories). Cells were photographed using a Leica laser

confocal microscope (Stellaris 8-STED Super-resolution, Germany) with a 60x oil lens and images were processed by ImageJ software.

1.2.9. Fluorescence polarization assay (FP)

These studies were conducted as previously reported [1, 2] using the labeled phosphopeptide, 5-carboxyfluorescein GpYLPQTV-NH_2 (where pY represents phospho-Tyr) as probe and purified recombinant Stat3, with some modifications. Briefly, for evaluating the effect of inhibitors on Stat3 binding to pY peptide, a fixed concentration of recombinant His-Stat3 protein (150 nM) was preincubated with 0-10 µM of unlabeled GpYLPQTV-NH_2 (positive control) or H182 at 30 °C for 30 min in the indicated assay buffer conditions, prior to the addition of the labeled probe at 10 nM final concentration and incubated for 30 min at room temperature, and thereafter reading the FP signal at 10, 30, and 60 min using the SpectraMax ID5 (Molecular Devices, San Jose, CA), with the set gain adjustment at 35 mP. In some cases, the recombinant His-Stat3 was pre-incubated with the unlabeled GpYLPQTV-NH_2 or H182 at 0-10 µM for 10 min or at 0-50 µM for 30 min prior to incubation with the labeled probe, and thereafter reading the FP signal at 10, 30, or 60 min using the SpectraMax ID5, with the set gain adjustment at 35 mP.

1.2.10. Isothermal titration calorimetry (ITC)

The ITC experiment was carried out as previously described [7, 8] with some modification using Malvern Panalytical MicroCal PEAQ-ITC (United Kingdom). Studies were done at 25 °C. Briefly, H182, previously suspended in 100 % DMSO were diluted in 20 mM HEPES, 150 mM KCl buffer so the final DMSO was 5 %. To avoid buffer mismatch, Stat3 in HEPES buffer was diluted in HEPES buffer with 5% DMSO final concentration. Three hundred microliter (300 µL) aliquots of 3.0 µM Stat3 were placed in the cell and titrated with 250 µM H182. Titrations took place by injecting 2 µL H182 in a 2.5 min injection for the titration peak to return to the baseline. The K_D was calculated using the MicroCal PEAQ-ITC analysis

software and Prism GraphPad software (San Diego, CA), using the one-site model. Control experiments were carried out by titration H182 into buffer, buffer into Stat3, and buffer into buffer. The three controls were used as a composite for the ITC experiment to subtract the heat of dilution and background noise from the measurements.

1.2.11. Wound healing assay for migration

Studies were performed as previously reported [1-3]. Briefly, cells were cultured in 6-well plates, and the monolayer of cells was scratched with a p10 pipet tip. Subsequently, fresh medium containing inhibitors or DMSO (control) was added, and then immediately, cells were imaged with a Canon Powershot A640 digital camera under a phase contrast with the Zeiss Axiovert 200 inverted microscope at 10X magnification and noted as the zero time-point. Following culture for 22 h to allow cells to migrate into the denuded area, the culture was then imaged again at the same field that was marked on the plate.

1.2.12. Annexin V binding/apoptosis and flow cytometric analysis

For apoptosis analysis, annexin V/propidium iodide (PI) staining was performed. Briefly, cells in culture and untreated (DMSO, control) or treated with H182 were harvested and stained with FITC-annexin V (Apoptosis Detection Kit), according to manufacturer's instructions (BD Biosciences, San Jose, CA). The annexin V- and/or PI-positive cells were analyzed by FACScan flow cytometer (BD Biosciences) and data was analyzed using FlowJo software.

1.2.13. Assessment of Physicochemical Properties

Solubility, human (HLM) and mouse liver microsomal (MLM), and plasma stability studies were performed by Eurofins Cerep Panlabs and Eurofin Discovery Services (www.eurofins.com/PharmaDiscovery), while

protein binding studies was performed by Sanford Burnham Prebys Chemical Biology & Drug Discovery Core using standard protocols.

1.2.1.4. Nano-LC/MS/MS analysi-

1.2.1.4.1. Sample preparation

Purified His-tagged recombinant wild type Stat3 protein containing residues 127 to 711 (wtStat3) [6] was incubated with compounds H098 or H182 at 2 μM each, or with DMSO as control for 1 h under the conditions that were used in the EMSA assay. Thereafter, proteins were precipitated with cold acetone at a final concentration of 80% at -20°C overnight. The samples were spun down and the pellets were washed with a large volume of cold methanol once and air-dried.

The in-solution proteolytic digestions were performed with three different enzymes separately. Trypsin, Chymotrypsin, and V8 were the enzymes selected to provide a large diversity in digested peptides for nano-LC/MS/MS analysis. Each enzyme digestion was performed at 1:100 enzyme to protein ratio and ran overnight (16 h) at 37 °C. Each completed digestion was acidified to a final concentration of 0.1% v/v formic acid, and speed vacuumed to dry pellet, and then reconstituted to 0.1% v/v formic acid readied for MS loading. Three enzyme-digested samples were finally combined as one for sample loading.

1.2.1.4.2. Orbitrap Fusion Loading and Run

Samples were loaded and desalted at a flow rate of 50 μl/min for 5 min on a C18 trap column (Waters Symmetry C18 180 μm x 2 cm) in acetonitrile at 2% v/v, water at 97.9% v/v, formic acid 0.1% v/v. The samples were separated on a C18 reversed-phase analytical column (Waters BEH C18 1.7 μm x 75 μm x 200 mm) using a Waters nanoAcquity UPLC over a 70 min gradient. Mobile phase A was 0.2% formic acid in water and mobile phase B was 0.2% formic acid in acetonitrile. The LC-gradient was the same as

previously described [9]. The Thermo Orbitrap Fusion mass spectrometer was used to analyze all samples using three sequential fragmentation methods (HCD, CID, ETD) in a DDA mode to maximize MS/MS spectra output. Peptide ions with *m/z* from 400-2000 were acquired in the Orbitrap with nominal resolution of 120k (FWHM) at m/z 200 for MS. Peaks above intensity 5e8 with charges 2-8 were selected for sequential CID, HCD, and ETD fragmentation in the Orbitrap at 30,000 resolution followed by dynamic exclusion for 15 s.

1.2.1.4.2. Bioinformatic Analysis

The raw MS data was processed using Proteome Discoverer to generate the MGF text files that were used in MASCOT database search against the Stat3 protein sequence. MASCOT search parameters were as following: no enzyme specified, precursor ion mass error 10 ppm, and fragment ion mass error at 0.1 Da. Custom modifications were included for the separate molecules (based on their mono-mass increase) used in the experiments. All PSMs were filtered with 5% FDR (expect value of 0.05 in MASCOT file).

2. Results

2.1. Medicinal chemistry/SAR-directed optimization of carboxylic acid-based lead Stat3 inhibitors

Through a thoughtful medicinal chemistry campaign, we have broken the micromolar potency barrier of the previous carboxylic acid-based Stat3 inhibitors [2, 3]. Per computer-guided strategy, we made modifications to the reported carboxylic acid-based leads, BP-1-102 [2], SH4-54 and SH5-07 [3]. Optimization included a key azetidine ring in the analogs, H105, H120, H172, and H182 (Fig. 1A). In analogs H172 and H182, phenyl is replaced by pyridine and in place of the carboxylic acid the bioisosteric 1-phthalazinone (H172) and 4-quinazolinone (H182) groups are present. (Fig. 1A). While all functionalities contributed to the enhanced potency and/or physicochemical properties, significantly, the azetidine moiety provided the greatest enhancement of Stat3-inhibitory potency. Details of the azetidine series of small

molecules, their chemistry and structure-activity relationship (SAR) studies appear in our recent publication [8].

3. References

[1] P. Yue, F. Lopez-Tapia, D. Paladino, Y. Li, C.-H. Chen, T. Hilliard, Y. Chen, M. Tius, J. Turkson, Hydroxamic acid and benzoic acid-based Stat3 inhibitors suppress human glioma and breast cancer phenotypes in vitro and in vivo, Cancer Res., 76 (2016) 652-663.

[2] X. Zhang, P. Yue, B.D. Page, T. Li, W. Zhao, A.T. Namanja, D. Paladino, J. Zhao, Y. Chen, P.T. Gunning, J. Turkson, Orally bioavailable small-molecule inhibitor of transcription factor Stat3 regresses human breast and lung cancer xenografts, Proc Natl Acad Sci U. S. A., 109 (2012) 9623-9628.

[3] C. Brotherton-Pleiss, P. Yue, Y. Zhu, K. Nakamura, W. Chen, W. Fu, C. Kubota, J. Chen, F. Alonso-Valenteen, S. Mikhael, L. Medina-Kauwe, M.A. Tius, F. Lopez-Tapia, J. Turkson, Discovery of Novel Azetidine Amides as Potent Small-Molecule STAT3 Inhibitors, J Med Chem, 64 (2021) 695-710.

[4] X. Zhang, P. Yue, S. Fletcher, W. Zhao, P.T. Gunning, J. Turkson, A novel small-molecule disrupts Stat3 SH2 domain-phosphotyrosine interactions and Stat3-dependent tumor processes, Biochem Pharmacol, 79 (2010) 1398-1409.

[5] K. Siddiquee, S. Zhang, W.C. Guida, M.A. Blaskovich, B. Greedy, H.R. Lawrence, M.L. Yip, R. Jove, M.M. McLaughlin, N.J. Lawrence, S.M. Sebti, J. Turkson, Selective chemical probe inhibitor of Stat3, identified through structure-based virtual screening, induces antitumor activity, Proc Natl Acad Sci U. S. A. 104 (2007) 7391-7396.

[6] K.A. Siddiquee, P.T. Gunning, M. Glenn, W.P. Katt, S. Zhang, C. Schrock, S.M. Sebti, R. Jove, A.D. Hamilton, J. Turkson, An oxazole-based small-molecule Stat3 inhibitor modulates Stat3 stability and processing and induces antitumor cell effects, ACS Chem Biol, 2 (2007) 787-798.

[7] A.T. Namanja, J. Wang, R. Buettner, L. Colson, Y. Chen, Allosteric Communication across STAT3 Domains Associated with STAT3 Function and Disease-Causing Mutation, J Mol Biol., 428 (2016) 579-589.

[8] F. Alonso-Valenteen, S. Pacheco, D. Srinivas, A. Rentsendorj, D. Chu, J. Lubow, J. Sims, T. Miao, S. Mikhael, J.Y. Hwang, R. Abrol, L.K. Medina Kauwe, HER3-targeted protein chimera forms endosomolytic capsomeres and self-assembles into stealth nucleocapsids for systemic tumor homing of RNA interference in vivo, Nucleic Acids Res, 47 (2019) 11020-11043.

[9] G. Drakakaki, W. van de Ven, S. Pan, Y. Miao, J. Wang, N.F. Keinath, B. Weatherly, L. Jiang, K. Schumacher, G. Hicks, N. Raikhel, Isolation and proteomic analysis of the SYP61 compartment reveal its role in exocytic trafficking in Arabidopsis, Cell Res, 22 (2012) 413-424.

Supplementary Figure Legends

Figure S1. **Fluorescence Polarization (FP) assay of the binding of the 5-carboxyfluorescein-GpYLPQTV-NH2 probe to purified recombinant His-Stat3**. Recombinant His-Stat3 was pre-incubated with the unlabeled peptide, GpYLPQTV-NH2 or H182 at (A) 0-10 μM for 10 min or (B) 0-50 μM for 30 min prior to incubation with the labeled probe for (i) 10 min, (ii) 30 min, or (iii) 60 min and recording the FP signals thereafter. Data are representative of four independent determinations

Figure S2. **Effects of azetidine-based compounds on Stat3 phosphorylation and DNA-binding activity in breast cancer cells.** (A) SDS-PAGE and immunoblotting analysis of whole-cell lysates of equal total protein prepared from MDA-MB-231 lines untreated (DMSO, 0, -) or treated 5 μM H105 or H120 for 0-24 h (i), or 0.5-3 μM H182 for 2 (ii) and probing for pYStat3, Stat3, pS727Stat3, GAPDH, or tubulin; and (B) Nuclear extracts of equal total protein prepared from MDA-MB-468 cells untreated (DMSO, 0) or treated with 5 μM H105, H120 or H182 for 0-3 h, were subjected Stat3 DNA-binding activity/EMSA analysis

using the hSIE probe that binds Stat3. Positions of Stat3:DNA complex or proteins in gel are labeled; control lane (0) represents nuclear extract or whole-cell lysate prepared from 0.5% DMSO-treated cells. Data are representative of 2-3 independent determinations.

Figure S3. **Azetidine-based compounds preferentially suppress anchorage-dependent growth and induce apoptosis of breast cancer cells harboring aberrantly-active Stat3.** (A, C) normal human mesothelial (A), and breast epithelial, MCF-10A or breast cancer, MCF-7 (C) cells that do not harbor constitutively-active Stat3, and (B) human breast cancer, MDA-MB-231 and MDA-MB-468 cells that do and are growing in 96-well culture were treated once with increasing concentrations of H105, H120, H172, or H182 for 72 h and the viable cell numbers were assayed using CyQuant cell proliferation kit and plotted as viable cell numbers (% of control) against concentration, from which IC_{50} values were derived; and (D) MDA-MB-231 cells treated with 1 or 3 μM H182 for 3 h were processed for Annexin V binding/flow cytometric analysis; control (Con, 0) represents samples from 0.5% DMSO-treated cells. Values, mean ± S.D., n=3-6. Data are representative of 3 independent determinations.

Figure S4. **H182 suppresses c-Myc, survivin, and vascular endothelial growth factor (VEGF) expression in breast cancer cells.** Immunoblotting analysis of whole-cell lysates of equal total protein prepared from the human breast cancer, MDA-MB-231 cells, untreated (DMSO, 0) or treated with H182 at 0.5-3 μM for 2 h (left panel) or 1 μM for 0.5-24 h (right panel) and probing for c-Myc, survivin, VEGF, GAPDH, or tubulin. Positions of proteins in gel are shown. Bands corresponding to the indicated proteins were quantified using ImageJ and calculated as percent of control (con), which are shown; control (0) lane represents whole-cell lysates prepared from 0.5% DMSO-treated cells. Data are representative of 2-3 independent determinations.

Figure S5. **H182 inhibits Stat3 DNA-binding activity and the expression of the Stat3 target genes, c-Myc, vascular endothelial growth factor (VEGF) and survivin in breast tumors *in vivo*.** (A) EMSA analysis of Stat3 DNA-binding activity in tumor tissue lysates of equal total protein prepared from untreated (Control, C) and treated (T) mice; and (B) Immunoblotting analysis of tissue lysates of equal total protein prepared from tumor tissues from untreated (Control, C) and treated (T) mice and probing for c-Myc, VEGF, survivin, and GAPDH. Positions of Stat3:DNA complexes or proteins in gel are shown.
